# A novel CSN5/CRT O-GlcNAc/ER stress regulatory axis in platinum resistance of epithelial ovarian cancer

**DOI:** 10.7150/ijbs.89700

**Published:** 2024-01-27

**Authors:** Tianqing Yan, Xiaolu Ma, Kaixia Zhou, Jiazhen Cao, Yanan Tian, Hui Zheng, Ying Tong, Suhong Xie, Yanchun Wang, Lin Guo, Renquan Lu

**Affiliations:** 1 Department of Clinical Laboratory, Fudan University Shanghai Cancer Center, No.270, Dong' An Road, Xuhui District, Shanghai 200032, China.; 2 Department of Oncology, Shanghai Medical College, Fudan University, Shanghai 200032, China.

**Keywords:** COP9 signalosome subunit 5, Calreticulin O-GlcNAcylation, Endoplasmic reticulum stress, Ovarian cancer, Platinum resistance

## Abstract

**Background:** High levels of COP9 signalosome subunit 5 (CSN5) in epithelial ovarian cancer (EOC) are associated with poor prognosis and are implicated in mediating platinum resistance in EOC cells. The underlying mechanisms, however, remained undefined. This study aimed to elucidate the molecular process and identify potential therapeutic targets.

**Methods:** RNA-sequencing was used to investigate differentially expressed genes between platinum-resistant EOC cells with CSN5 knockdown and controls. O-GlcNAc proteomics were employed to identify critical modulators downstream of CSN5. The omics findings were confirmed through qRT-PCR and immunoblotting. In vitro and in vivo experiments assessed the sensitivity of resistant EOCs to platinum.

**Results:** We demonstrated an involvement of aberrant O-GlcNAc and endoplasmic reticulum (ER) stress disequilibrium in CSN5-mediated platinum resistance of EOC. Genetic or pharmacologic inhibition of CSN5 led to tumor regression and surmounted the intrinsic EOC resistance to platinum both in vitro and in vivo. Integration of RNA-sequencing and O-GlcNAc proteomics pinpointed calreticulin (CRT) as a potential target of aberrant O-GlcNAc modification. CSN5 upregulated O-GlcNAc-CRT at T346 to inhibit ER stress-induced cell death. Blocking T346 O-GlcNAc-CRT through CSN5 deficiency or T346A mutation resulted in Ca^2+^ disturbances, followed by ER stress overactivation, mitochondrial dysfunction, and ultimately cell apoptosis.

**Conclusion:** This study reveals that CSN5-mediated aberrant O-GlcNAc-CRT acts as a crucial ER stress checkpoint, governing cell fate response to stress, and emphasizes an unrecognized role for the CSN5/CRT O-GlcNAc/ER stress axis in platinum resistance of EOC.

## 1. Introduction

Platinum resistance is a primary cause of recurrence in epithelial ovarian cancer (EOC) 1. Presently, understanding of the critical molecular mechanisms influencing the response of OC patients to platinum-based chemotherapy remains limited. Among the chemotherapy platinum drugs, cisplatin stands out as one of the most effective treatments for ovarian cancer 2. Given its substantial impact on patients' prognosis, enhancing the response to platinum-based chemotherapy in platinum-resistant or refractory OC represents a significant challenge.

O-linked beta-N-acetylglucosamine (O-GlcNAc) glycosylation is a common post-translational modification in eukaryotes, predominantly occurring on serine and threonine residues. This modification is synthesized mainly through the hexosamine biosynthetic pathway (HBP), which is a branch of glucose metabolism, and governed by O-GlcNAc transferase (OGT) and O-GlcNAcase (OGA), enzymes that respectively add and remove O-GlcNAc from proteins 3. Previous studies have demonstrated that O-GlcNAcylation is integral to various diseases such as diabetes, cardiovascular disorders, neurodegenerative conditions, and cancer, making it an appealing target for disease diagnosis and therapy 4-6. Additionally, the glycosylation state of tumor regulatory factors impacts the malignant phenotype, material and energy metabolism, and epigenetic characteristics of tumors 7. The endoplasmic reticulum (ER), a crucial site for protein folding and an intracellular Ca^2+^ reservoir, is highly sensitive to cellular homeostasis changes 8. Disruptions in the protein folding environment can cause aggregated unfolded or misfolded proteins in the ER, initiating ER stress responses that can lead to either cellular survival or destruction 9-11. Calreticulin (CRT), a primary soluble Ca^2+^-binding glycoprotein in ER, facilitates molecular chaperone and Ca^2+^ homeostasis regulation within the ER, and outside of it, regulates numerous intracellular signals, including apoptosis 12, 13. Moreover, CRT activation has been linked with ER stress 14. Interestingly, the O-GlcNAcylation of CRT was first identified in the brains of patients with mild cognitive impairment and Alzheimer's disease in 2010^15^, but its specific role in EOC's platinum resistance remains undefined.

Our group previously detected constitutively photomorphogenic 9 signalosome subunit 5 (CSN5) to be significantly elevated in tissues of platinum-resistant patients and in in vitro developed platinum-resistant EOC cells (DDP cells), correlating with poor EOC prognosis 16. Inhibiting CSN5 genetically or pharmacologically was found to boost the sensitivity of DDP cells to platinum treatment 16. In the current study, RNA-sequencing and O-GlcNAc proteomics technologies were used to uncover aberrant CRT O-GlcNAc and ER stress as factors in the CSN5-mediated downstream regulation of platinum resistance in EOCs. Research on the impact of O-GlcNAc-CRT on EOC's platinum resistance is still in the nascent stage. We posit that CSN5-mediated O-GlcNAcylation of CRT may act as a vital safeguard for the correct protein folding and normal physiological functions in the ER. The specific mechanism by which the genetic or pharmacologic ablation of CSN5 renders DDP cells sensitive to platinum was elucidated, offering a fresh perspective for the treatment of platinum resistance.

## 2. Materials and methods

### 2.1 Tissue samples

Cancerous tissues of EOC patients during 2014-2018 who received tumor debulking surgery were collected from Fudan University Shanghai Cancer Center (FUSCC). This study was approved by the FUSCC Research Ethics Committee. Written informed consent was available from all patients. The inclusion and exclusion criteria could be referred to the previous publication 17.

### 2.2 Cells and reagents

The ovarian cancer cell lines A2780 and SKOV3 were obtained from American Type Culture Collection (Manassas, VA, USA), and the cisplatin-resistant clones A2780/DDP, SKOV3/DDP were established in our own lab as previously reported 18. All cells were cultured in Dulbecco's modified Eagle's medium (DMEM; Gibco, Carlsbad, USA) with 10% (v/v) fetal bovine serum (FBS; Gibco, Carlsbad, USA). The 4-phenylbutyric acid (4-PBA) was purchased from Selleck Co., Ltd (China, Catalog No.: S4125). The CSN5i-3 for CSN5 inhibition was obtained from MedChemExpress Co., Ltd (China, Catalog No.: HY-112134). OGA inhibitor-PUGNAc were borrowed from Sigma Co., Ltd (Catalog No.: A7229, USA).

### 2.3 Lentivirus-mediated shRNA infection and lipofectamine-mediated siRNA transient transfection assay

Cell lines with stable CSN5 down-expression and its control counterparts were established by lentivirus infection. The transfection procedure followed the manufactures' instructions. Specifically, DDP cells were seeded at a density of 1 × 10^5^ per well in six-well plates and infected with lentivirus. Following overnight infection, the medium was replaced with 2 µg/mL of puromycin and incubated for at least three days until the uninfected cells died. For siRNA transient transfection, DDP cells in six-well plates were incubated with 2.5 µg of siRNA (GenePharma, China) with 6 µL of lipofectamine 2000 (Invitrogen, USA). Cells were used for subsequent experiments. The shRNA and siRNA sequences used in this study were enlisted in **Table S1, 2**.

### 2.4 Colony-formation assay

Cells were seeded into 6 well-plates at a density of 5×10^3^ cells/well. After inherence, cells were treated accordingly and incubated for 24 h. Then, cells' medium was changed into fresh one and continue culturing for 10-14 days until visible clones appeared. Finally, cell colony was stained with 0.5% crystal violet and pictures were taken under good light.

### 2.5 Cell counting kit-8 (CCK-8) assay

Cells were seeded into 6 well-plates at a density of 1×10^4^ cells/well. After appropriate treatment, the CCK-8 reagent (Yeasen Biotechnology (Shanghai) Co., Ltd.) was mixed with cell culture medium in 1:10, and the mixture was added into each well and incubated for 2 h at 37℃. Finally, the microplate reader (Bio-Tek, Winooski, VT, USA) was employed to measure the optical density (OD) at 450 nm.

### 2.6 Cellular apoptosis experiments

Cells were seeded at a density of 2 × 10^5^ per well in six-well plates and treated accordingly. Cells were harvested and washed twice with PBS. Apoptosis was detected by staining the cells with Annexin V and 7-AAD according to the instructions indicated in the PE Annexin-V Apoptosis Detection Kit I (559763, BD Biosciences, USA). Stained cells were analyzed using Cyto-FLEXS (Beckman Coulter, USA) or photographed under a microscope (Olympus IX73, magnification × 100).

### 2.7 RNA-sequencing

Cell lines with stable CSN5 down-expression and its control counterparts were transfected into DDP cells, and the total RNA was extracted using TRIzol. RNA sequencing and data analysis were performed by Jie Yi Biotechnology Co., Ltd (Shanghai, China). The analysis was carried out by “limma” R package and the significance threshold was set as |log_2_[fold change (FC)] | > 1, and false discovery rates (FDR) < 0.05. Gene ontology and KEGG pathway enrichment figures were established using the online database https://www.chiplot.online/, accessed on May 2023.

### 2.8 O-GlcNAc proteomics

O-GlcNAc modified glycoproteins were specifically enriched using anti-O-GlcNAc antibodies by immunoprecipitation. The protein samples were separated by SDS-PAGE and stained using Coomassie bright blue reagent. The fresh protein strip gel was cut and shredded and placed in 96-well microplates. The chopped colloidal particles were first decolorized with 200 μL decolorizing solution, then washed three times until the colloidal particles were transparent, and then dehydrated and dried twice with 200 μL acetonitrile. The dried and dehydrated colloidal granules were added to digestive solution (25 mM NH_4_HCO_3_ solution containing 0.01 μg/μL trypsin) and incubated for 20 min. The colloidal granules were covered with 25 mM NH_4_HCO_3_ solution and transferred to 37℃ for incubation and digestion overnight. Finally, 200 μL extract solution (50% acetonitrile solution containing 0.1% trifluoroacetic acid) was extracted twice, and the polypeptides in the supernatant were collected and merged. The extract was dried under N_2_ protection. The test was performed using the Thermoelectric Easy-nLC 1200 (Thermo Scientific, P/N LC140) and Orbitrap Exploris 480 (Thermo Scientific, P/N BRE725533) instruments. Gene ontology and KEGG pathway enrichment figures were established using the online database https://www.chiplot.online/, accessed on May 2023.

### 2.9 Measurement of cytosolic and mitochondrial Ca^2+^ levels

The cytosolic Ca^2+^ levels were detected using Fluo-8 probe (APPLYGEN, C0012, China) according to the manufactures' protocol. Briefly, cells were seeded at 1×10^4^ cells/100 µL/well in 96- or 100×10^4^ cells/2000 µL/well in 60 mm dishes, and cultured in cell incubator. After appropriate treatments, discard the culture medium, add the Fluo-8 working solution to cover the cells, and incubate at 37℃ for 30 min. Finally, cells were washed twice with dilution buffer, and then photographed under a microscope (Olympus IX73, magnification × 100) or cells can be collected and measured by Cyto-FLEX S (Beckman Coulter, USA) using FITC channels. For the mitochondrial Ca^2+^ examination, either Rhod-2 AM (MedChemExpress, HY-D0989, China) or Fluo-8 probe plus Mito-tracker reagent (C1035, Beyotime, China) was employed following the manufactures' instructions.

### 2.10 Determination of intracellular reactive oxygen species (ROS)

Cells were seeded at a density of 2 × 10^5^ per well in six-well plates and treated appropriately. The ROS analysis was performed by diluting dihydroethidium (S0063, Beyotime, China) with a pre-heated complete medium to 5 μM and incubating cells at 37 °C in the dark for 20 min. Finally, cells were analyzed and quantified using Cyto-FLEX S (Beckman Coulter, USA).

### 2.11 Detection of mitochondrial membrane potential (MMP)

Mitochondrial membrane potential was evaluated using JC-1 Mitochondrial Membrane Potential Assay Kit (Med Chem Express, HY-K0601) according to the manufactures' instructions. Briefly, cells were resuspended in PBS at 1×10^6^ cells/mL. JC-1 was added to a final concentration of 2 μmol/L. After that, cells were incubated at 37℃ for 20 min before flowcytometry analysis (Cyto-FLEX S, Beckman Coulter, USA).

### 2.12 Co-Immunoprecipitation and immunoblotting

Cells were scraped following treatment and the whole-cell protein was extracted with NP40 lysis buffer (P0013F, Beyotime, China) supplemented with protease inhibitors. Protein concentrations were measured using a BCA Protein Quantification Kit (20201ES76, YEASEN, China). The whole-cell protein was incubated with the relevant antibody or the control IgG (2729, CST, USA) overnight at 4 °C. The next day, protein A/G magnetic beads (HY-K0202-1, MCE) were added into the mixture for 2.5 h at 4 °C. Subsequently, the beads were washed five times with PBST (1×PBS + 0.5% Tween-20, pH 7.4) and were boiled. After that, the protein samples were separated electrophoretically and transferred to polyvinylidene fluoride membranes (ISEQ00010, Merck Millipore, USA). Then, 5% non-fat milk (A600669, Sangon Biotech, China) was used for blocking. The primary and secondary antibodies used in this study were listed in **Table S3.**

### 2.13 Cycloheximide chasing assay

Cells with CSN5 inhibition or its counterparts were seeded at a density of 2 × 10^5^ per well in six-well plates and treated with cycloheximide (CHX; MedChemExpress, HY-12320; 50 μg/mL) for 0 to 12 h. Cell lysates were then prepared by using a lysis buffer containing 2% SDS, followed SDS-PAGE and immunoblotting analysis with specific antibodies.

### 2.14 CRISPR-Cas9 genome editing-mediated knockout (KO) of CRT

The CRISPR-Cas9 plasmid vectors were constructed by Shanghai Genechem Co.,Ltd. The hU6-sgRNA-CBA promotor-3Flag-Cas9-T2A-puro (GV465) plasmid was used as a delivery system. Single guide RNA (sgRNA) sequences targeting CRT and a scrambled sgRNA** (Table S4)** were cloned into the BsmBI restriction site of the GV465 plasmid. The plasmids were stably transfected into A2780/DDP cells, and the cells were screened with puromycin (2 mg/mL) for 1-2 weeks. Immunoblotting was employed to confirm the KO efficiency of CRT.

### 2.15 Quantitative real-time fluorescent polymerase chain reaction (qRT-PCR)

Total RNA was isolated using the RNA-Quick Purification Kit (RN001, ES Science, China) and then reversely transcribed into cDNA using a Prime Script RT Master Mix (RR036A, TAKARA, Japan). The quantitative real-time PCR was carried out by Hieff UNICON® Universal Blue qPCR SYBR Green Master (11184ES03, Yeasen, China) using the QuantStudioTM DX system (Thermo Fisher Scientific, USA). The relative amount of each gene was calculated using the 2^-ΔΔCt^ method and normalized to the Glyceraldehyde-3-phosphatedehydrogenase (GAPDH). **Table S5** enlisted the primers sequences used in the present study.

### 2.16 Immunofluorescent staining

Cells were seeded in 24-well dishes or confocal dishes and exposed to treatments accordingly. Then, cells were washed with PBS and fixed with 4% of paraformaldehyde, followed by permeabilization using 0.3% of Triton X-100. Cells were next washed and blocked with a 5% bovine serum albumin blocking buffer in room temperature for 1 h. Subsequently, primary antibody was added into cells overnight at 4 ℃. On the next day, cells were washed and incubated with the corresponding fluorescent secondary antibody in the dark. Afterwards, cells were incubated with fluorescence quench- preventing solution (including DAPI, Beyotime, P0131) in the dark. Photos were taken under the ordinary inverted fluorescence microscope (Olympus IX73) or the laser scanning confocal microscope (Leica STELLARIS DMi8).

### 2.17 *In vivo* xenotransplant assay

All animal experiments followed the protocols authorized by the Research Ethics Committee of FUSCC. Female BALB/C mice (4-6 weeks old, 16-18 g) were purchased from Beijing Life River Laboratory Animal Technology and raised under pathogen-free conditions. To investigate the effect of CSN5 signaling on EOC platinum resistance *in vivo*, 1 × 10^7^ cells were injected into the flanks of mice. To assess *in vivo* resistance, tumor-bearing mice were randomly divided and receiving treatments accordingly. Tumor size and body weight of mice were measured every 2 days. The calculation formula of tumor volume is: 0.5 × L × W^2^, where L is the tumor size at the longest point and W is the tumor size at the widest point. Finally, animals were euthanized by CO_2_ inhalation anesthesia and tumors were removed for subsequent experiments.

### 2.18 Immunohistochemistry

Formalin-fixed paraffin-embedded tissue sections were placed in a 60℃ incubator for 1-2 h, and subsequently were dewaxed with xylene and then rehydrated with ethanol. Samples were boiled in EDTA buffer (pH 9.0) for antigen repair. Endogenous peroxidase was treated with 3% catalase for 10 min at room temperature. Then samples were blocked at room temperature for 1 h and incubated with primary antibody at 4°C overnight. The next day, biotinylated goat anti-rabbit/mouse secondary antibody (Shanghai Chang Dao Antibody) was incubated at room temperature for 30 min, washed and colored with DAB dye solution for 5 min. Finally, the sections were counterstained with hematoxylin, dehydrated and sealed, and photographed under an inverted fluorescence microscope (Olympus IX73).

### 2.19 Statistical analysis

SPSS (V.25.0, IBM, Armonk, New York, NY, USA) and GraphPad Prism v8 (GraphPad Software, Inc., SanDiego, CA, USA) were used for mapping and statistical analyses. Data were presented as mean ± standard error. Student's t test was used to compare two groups, and ANOVA was used to compare multiple groups. Correlation between two groups was analyzed using nonparametric Spearman's r test.* p* < 0.05 was considered statistically significant.

## 3. Results

### 3.1 CSN5 inhibition sensitizes EOC to platinum in vitro and in vivo and induces apoptosis in platinum-resistant EOC cells

**Figure 1:**
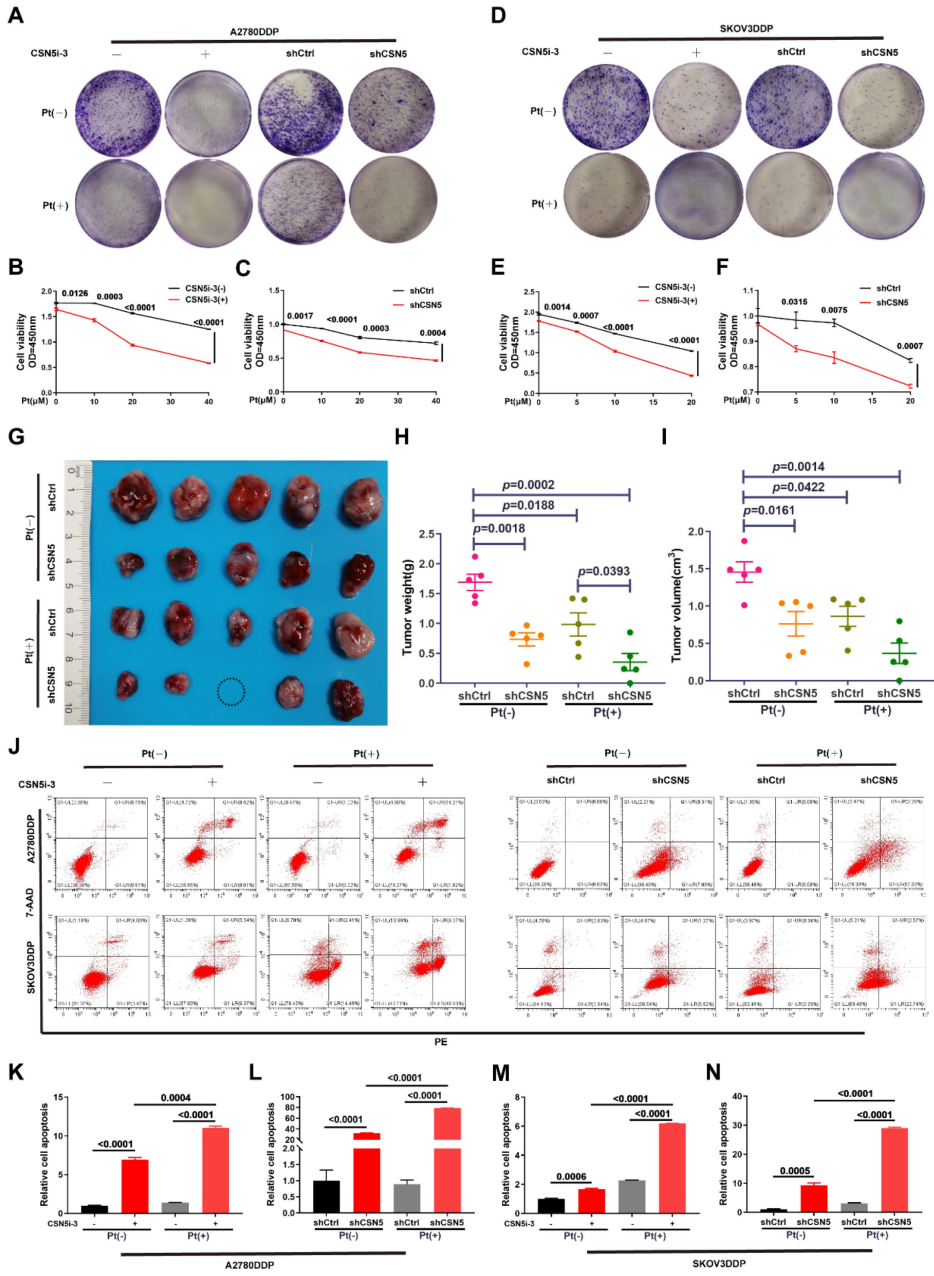
** CSN5 inhibition sensitizes EOC to platinum in vitro and in vivo and induces apoptosis in platinum-resistant EOC cells.** (A, D) DDP cells with or without CSN5 inhibition, genetically or pharmacologically, were exposed to cisplatin or not for 24 h (CSN5i-3, 5 μM; Cisplatin, 20 μM, A2780DDP; 10 μM, SKOV3DDP). The proliferation effects were assessed by colony formation assay. (B, C, E, F) DDP cells with or without CSN5 inhibition were treated with different doses of cisplatin for 24 h, and the cellular viability was evaluated by CCK-8 assay. (G) An in vivo xenograft model was employed to show the anti-tumor effects of CSN5 inhibition (Cisplatin, 5 mg/kg i.p). (H, I) Tumor weight and volume were analyzed. (J) Annexin V-FITC/7-AAD staining was employed to test the cellular apoptosis of DDP cells with or without CSN5 inhibition. (K-N) Flow cytometry was used to detect and quantify the cellular apoptotic levels.

Our prior research has confirmed that inhibition of CSN5 is sufficient to suppress the malignant phenotypes of DDP cells 16. In this study, we used colony formation and CCK-8 assays to demonstrate that the combination of CSN5 inhibition with platinum treatment significcantly inhibited the propagation viability of DDP cells (**Figure 1A‑F**). To further elucidate the role of CSN5 in platinum-resistant EOC, we conducted in vivo xenograft experiments using nude mice. A2780/DDP cells with CSN5 inhibition, or their counterparts, were subcutaneously injected into the right flank of nude mice. Cisplatin was administered intraperitoneally twice a week for one month. As depicted in **Figure 1G-I**, knockdown of CSN5 markedly inhibited tumor growth in the nude mice. Mice with tumors treated with CSN5 knockdown followed by platinum exhibited even smaller tumor sizes than those treated with CSN5 inhibition alone. These findings suggest that both in vitro and in vivo tumor growth can be inhibited, and that the addition of cisplatin further amplifies these effects. Additionally, cellular apoptosis was evaluated through flow cytometry (FCM) analysis. Apoptosis rates were found to increase with genetic or pharmacological CSN5 inhibition, and cisplatin intervention further augmented the apoptotic effects (**Figure 1J-N**).

### 3.2 Protein O-GlcNAcylation participates in CSN5-mediated platinum resistance of ovarian carcinoma

**Figure 2:**
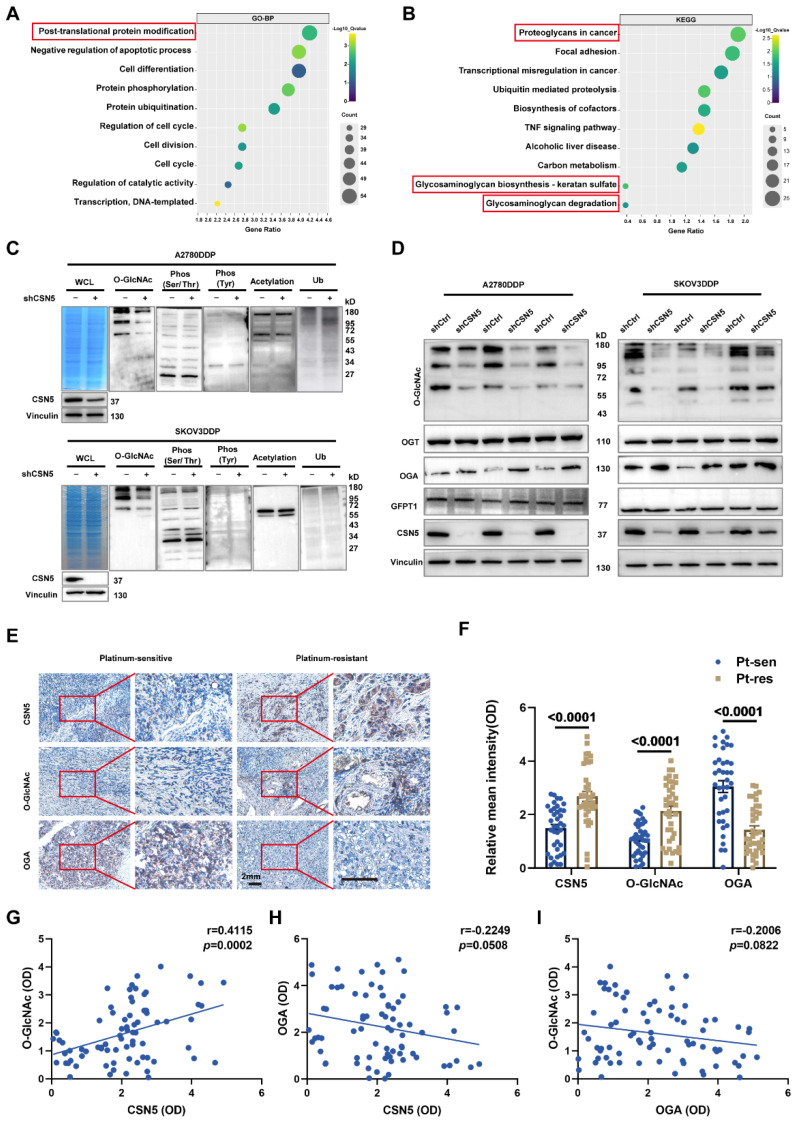
** Protein O-GlcNAcylation participates in CSN5-mediated platinum resistance of ovarian carcinoma.** (A, B) GO and KEGG enrichment analysis of upregulated DEGs (fold change > 1) in platinum-resistant EOC cells after CSN5 knockdown based on the RNA-seq results. (C) IB analysis was used to analyze the changes in the whole protein post-translational modification levels under different CSN5 expression levels, including O-GlcNAcylation, ubiquitination, phosphorylation (Ser/Thr/Tyr), and acetylation. (D) DDP cells with or without CSN5 knockdown were treated with cisplatin for 24 h. Cells were lysed and total proteins were collected to detect the levels of protein O-GlcNAcylation, OGT, OGA, and GFPT1 by IB analysis. (E) Representative IHC staining intensities of CSN5, O-GlcNAc, and OGA between platinum-sensitive and resistant ovarian cancer tissues (n = 76, Scale bar = 2 mm). (F) Quantification and statistical analysis of the IHC staining results. (G-I) The IHC staining intensities of CSN5, protein O-GlcNAc, and OGA were applied to Spearman correlation analysis, and the points represent the number of patients involved (n = 76).

To elucidate the mechanism underlying the CSN5 inhibition-mediated enhancement of chemotherapeutic sensitivity in EOC, an initial RNA-sequencing was conducted to identify the differential expression genes (DEGs) between DDP cells with CSN5 knockdown and corresponding controls. Gene ontology (GO) analysis demonstrated that most of the DEGs following CSN5 silencing pertained to protein post-translational modification (PTM) (**Figure 2A**). Analysis via the Kyoto Encyclopedia of Genes and Genomes (KEGG) indicated that pathways tied to cancer proteoglycans and glycosaminoglycan metabolism were markedly enriched after CSN5 knockdown (**Figure 2B**). Next, to investigate potential functional PTMs in CSN5-mediated platinum resistance of EOC, immunoblotting (IB) was employed to screen various common modification types, as informed by functional enrichment analysis. These included O-GlcNAcylation, phosphorylation (Ser/Thr/Tyr), acetylation, and ubiquitination. The results revealed that protein O-GlcNAcylation was notably diminished (**Figure 2C**). Additionally, a reduction of O-GlcNAc in the CSN5 knockdown group was strongly correlated with an increase in OGA expression, while levels of OGT and the rate-limiting enzyme GFPT1 of the HBP pathway remained unchanged post-CSN5 inhibition (**Figure 2D and Figure S1 and S2**). IHC staining was utilized to further substantiate the expressions of CSN5, O-GlcNAc, and OGA in tissues from platinum-sensitive and resistant ovarian cancer patients. In contrast to platinum-sensitive tissues, levels of CSN5 and O-GlcNAc were pronounced in platinum-resistant EOC tissues, while OGA expression was minimally distributed (**Figure 2E, F**). Correlation analyses unveiled a positive relationship between CSN5 and protein O-GlcNAc, and a negative correlation with OGA (**Figure 2G, H**). Levels of protein O-GlcNAc showed a negative correlation trend with OGA, though the difference was not statistically significant (**Figure 2I**). These findings suggest that CSN5 deletion in DDP cells might suppress overall protein O-GlcNAcylation through the upregulation of OGA expression.

### 3.3 CSN5 protects platinum-resistant EOC cells from platinum injury by regulating ER stress signaling

**Figure 3:**
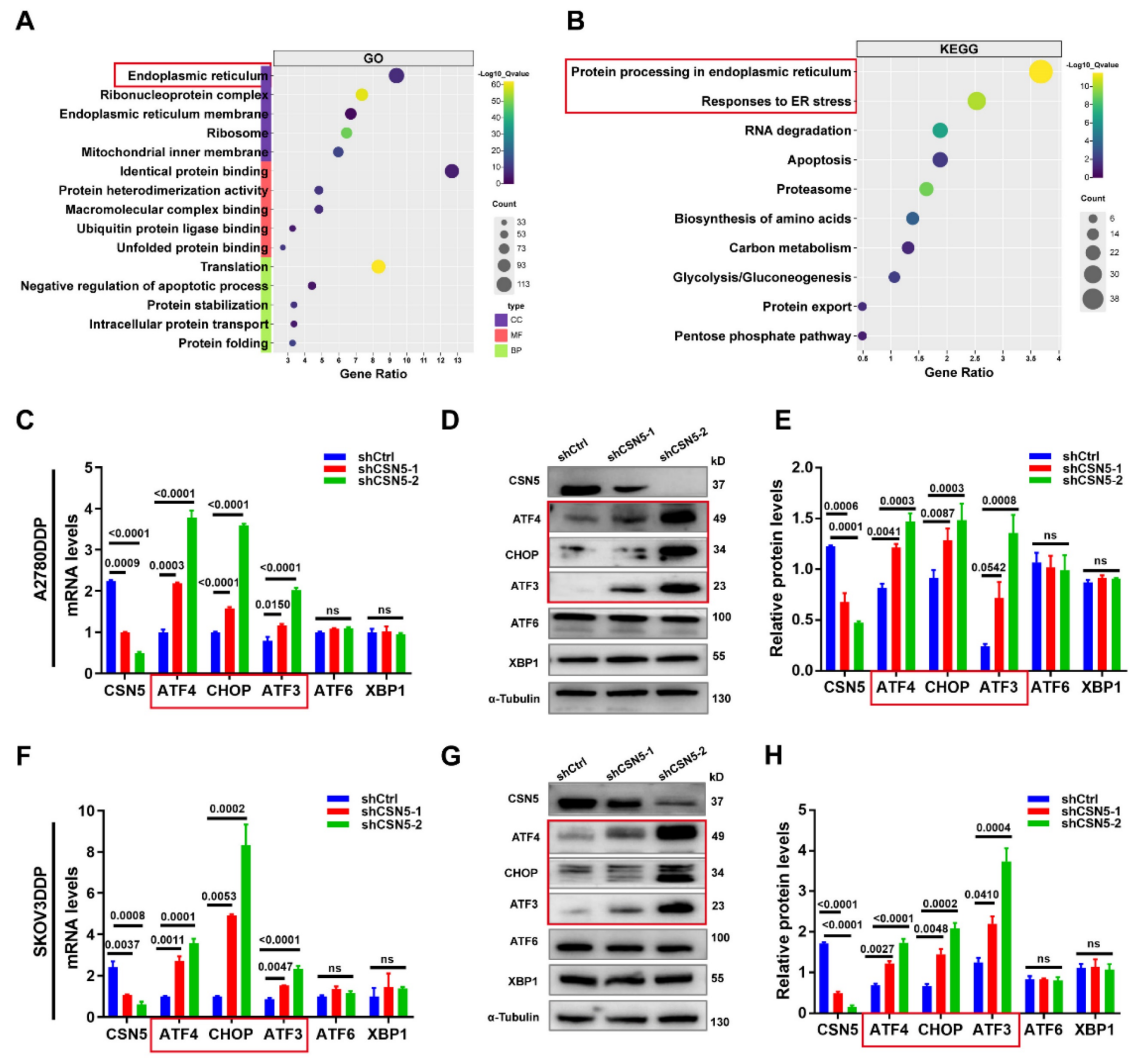
** CSN5 protects platinum-resistant EOC cells from platinum injury by regulating ER stress signaling.** Proteomic analysis was applied to identify the O-GlcNAcylated proteins in platinum-resistant EOC cells with or without CSN5 knockdown, and functional characterization of the identified O‑GlcNAc proteins was analyzed. (A, B) Significantly enriched biochemical and signaling pathways of the identified O-GlcNAcylated proteins were revealed by integrated GO and KEGG pathways analysis (*p* < 0.05). (C, F) qRT-PCR analysis of the representative gene expression contained in the ER stress signaling pathway in DDP cells with or without CSN5 inhibition. (D, G) IB analysis of the representative ER stress signaling protein levels. (E, H) Densitometric analysis of protein expression contained in the ER stress signaling pathway. Experiments were performed in triplicate.

To identify the critical player and potential regulatory pathways associated with O-GlcNAcylated protein in platinum-resistant EOC cells, these proteins were chemo-enzymatically enriched and then characterized using liquid chromatography in tandem with mass spectrometry (MS). Gene ontology analysis revealed that O-GlcNAcylated proteins in the CSN5 knockdown group were primarily located in the ER (**Figure 3A**), with the most enriched functional terms relating to responses to ER stress (**Figure 3B**). Subsequently, qRT-PCR and IB methods were employed to validate the genes related to ER stress. As depicted in **Figure 3C-H**, both mRNA and protein levels of C/EBP-homologous protein (CHOP) and activating transcription factor 3 (ATF3) were elevated following CSN5 deletion. In addition, ATF4, an upstream molecule of CHOP, was also upregulated. Conversely, ATF6 and X-box binding protein 1 (XBP1), the other two upstream molecules of CHOP, remained unaltered post-CSN5 intervention (**Figure S3**). This observation indicates that the ATF4-CHOP pathway might be involved in the sensitivity of EOC to CSN5 inhibition-mediated chemotherapy.

### 3.4 Ca^2+^ disequilibrium and mitochondrial dysfunction occur in CSN5 suppressed platinum-resistant EOC cells

**Figure 4:**
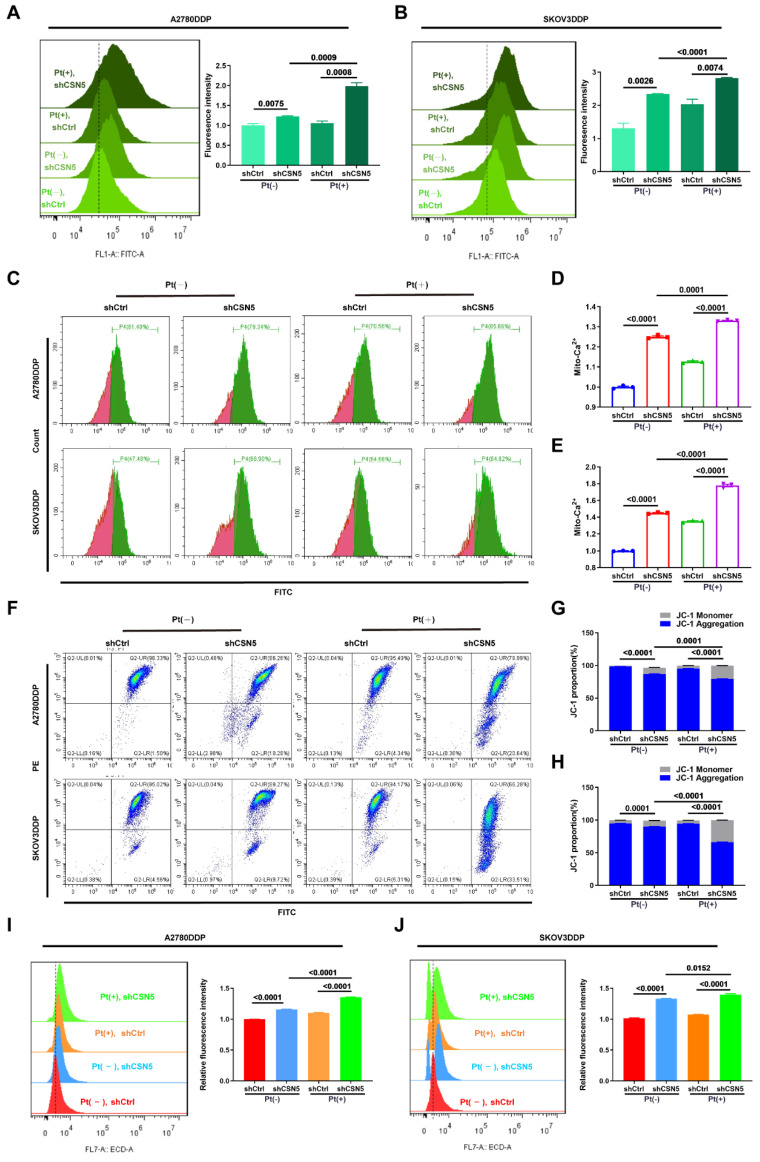
** CSN5 inhibition induced Ca^2+^ disturbances and mitochondrial dysfunction.** DDP cells with or without CSN5 knockdown were exposed to cisplatin or not for 24 h (Cisplatin, 20 μM, A2780DDP; 10 μM, SKOV3DDP). (A-E) Flow cytometry was used to examine the cytoplasmic and mitochondrial Ca^2+^ levels, and the relative Ca^2+^ levels were represented as a bar graph in relative percentage-ratio under the flow cytometry data. (F) The disruption of MMP was investigated using the JC-1 assay. (G, H) The relative JC-1 red/green ratio is represented as a bar graph in relative percentage-ratio under the flow cytometry data. (I, J) ROS levels were investigated using a DHE-labeled kit. Relative cellular ROS levels were represented as a bar graph in relative percentage-ratio under the flow cytometry data. All experiments were performed in triplicate.

It has been reported that an imbalance in Ca^2+^ homeostasis often accompanies mitochondrial dysfunction, leading to disturbances in ER stress 19, 20. When internal environmental homeostasis is compromised, the ER and mitochondria interact to induce apoptosis 21, 22. This ER stress within cells triggers the release of a substantial amount of Ca^2+^ into the cytoplasm, opening the mitochondrial permeability transition pore and causing mitochondrial swelling and the activation of the mitochondrial apoptosis pathway. Simultaneously, disruption of Ca^2+^ homeostasis in the ER activates ER stress coping responses 23, 24. To explore this, the intracellular Ca^2+^ concentration was examined following CSN5 downregulation. FCM analysis (**Figure 4A-E**) revealed that Ca^2+^ accumulated in the cytoplasm and mitochondria in DDP cells under CSN5 inhibition, an effect that was amplified when combined with cisplatin. Immunofluorescent staining tests also confirmed this result (**Figure S4A-C**). Furthermore, mitochondrial function was assessed, including investigations into mitochondrial membrane potential (MMP) and ROS production. The FCM analysis and immunofluorescent staining results suggested a decrease in MMP (**Figure 4F-H and Figure S4D-F**) and an increase in ROS production (**Figure 4I, J and Figure S4G, H**) following CSN5 inhibition, either genetically or pharmacologically, with cisplatin intervention exerting more obvious effects.

### 3.5 Increased susceptibility of platinum-resistant EOC cells mediated by CSN5 deficiency can be partly abrogated by ER stress suppressor

**Figure 5:**
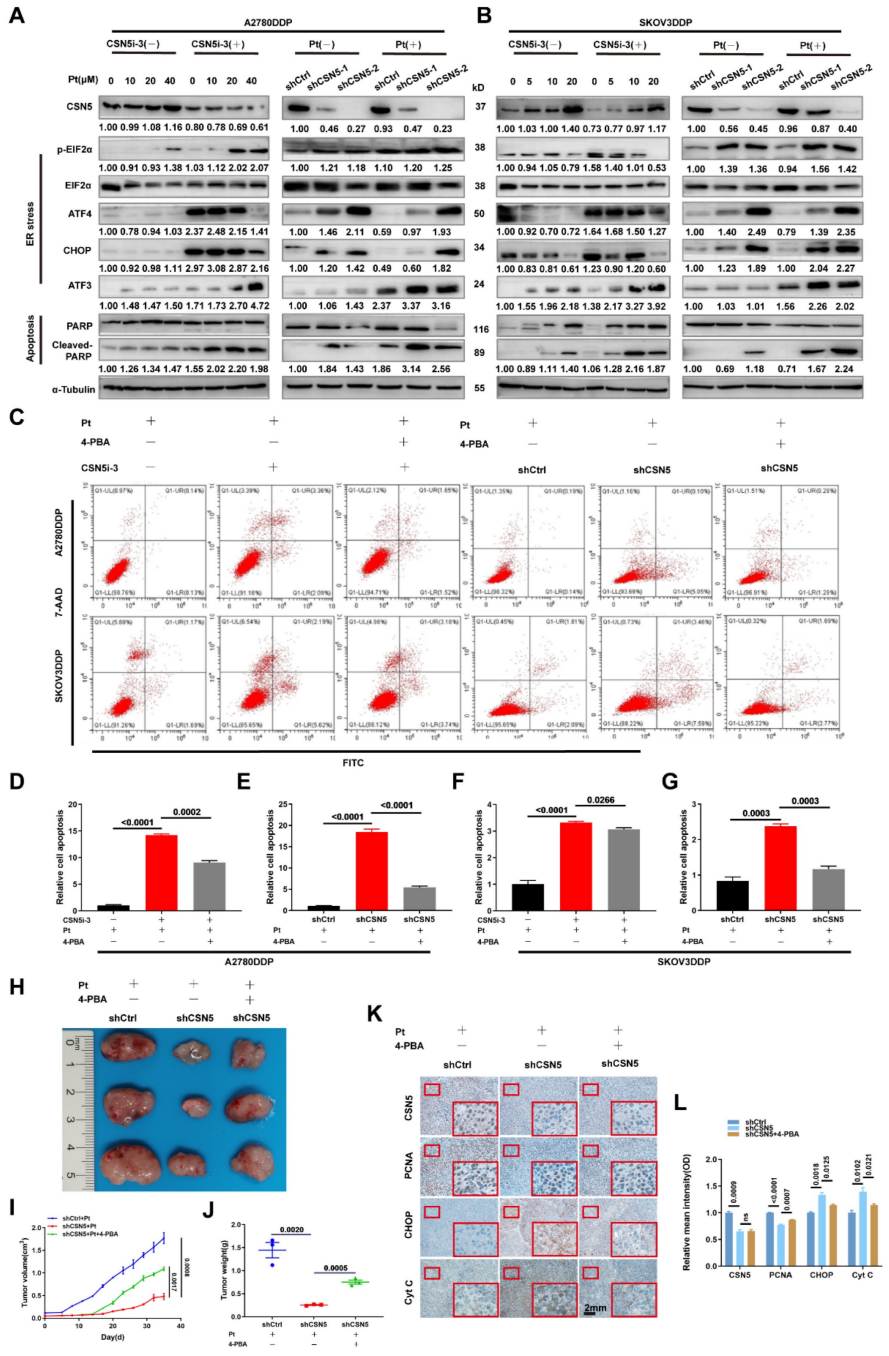
** Increased susceptibility of platinum-resistant EOC cells mediated by CSN5 deficiency can be partly abrogated by ER stress suppressor.** (A, B) Markers of ER stress and apoptosis in immunoblots were analyzed in DDP cells with or without CSN5 inhibition at different doses of cisplatin for 24 h. (C-G) DDP cells with or without CSN5 inhibition exposed to cisplatin were treated with 4-PBA (an ER stress signaling suppressor, 10 μM) or not. Flow cytometry was used to detect and quantify the cellular apoptotic levels. (H) An in vivo xenograft model showed the antagonistic action of 4-PBA on the anti-tumor effects mediated by CSN5 inhibition. (I, J) Tumor weight and volume were analyzed. (K) IHC results exhibited the proliferative marker PCNA, ER stress marker CHOP, and the apoptotic marker Cyt C (Scale bar = 2 mm). (L) The IHC staining intensity was quantified and shown as mean ± SEM.

We have demonstrated that ER stress was initiated, leading to the activation of downstream apoptosis following CSN5 inhibition. Subsequently, the ER stress signaling pathways and corresponding downstream apoptotic markers were assessed by IB. The results revealed that the expression of ER stress signaling proteins and the apoptotic marker, cleaved-PARP, were elevated after CSN5 knockdown or pharmacological inhibition when treated with varying doses of cisplatin (**Figure 5A, B**). Furthermore, the apoptotic responses induced by CSN5 inhibition in DDP cells could be partially abrogated by intercepting ER stress signaling (through 4-PBA supplementation or siRNA-mediated inhibition of CHOP) or by involving a caspase suppressant (Z-VAD-FMK) (**Figure 5C-G and Figure S5**). In vivo xenograft experiments provided evidence that tumor growth inhibition facilitated by CSN5 deficiency could be alleviated after 4-PBA intervention (**Figure 5H-J**). Additionally, IHC staining demonstrated a decrease in the proliferative marker PCNA, while ER stress and apoptotic markers CHOP and Cyt C were elevated in tumor cells subjected to CSN5 inhibition (**Figure 5K, L**), and 4-PBA involvement could reverse the trend. These findings suggest that CSN5 inhibition at least partially nullifies the resistance of DDP cells to platinum-induced apoptosis through the activation of ER stress signaling.

### 3.6 CSN5 downregulation stabilizes OGA, modulating CRT O-GlcNAcylation

**Figure 6:**
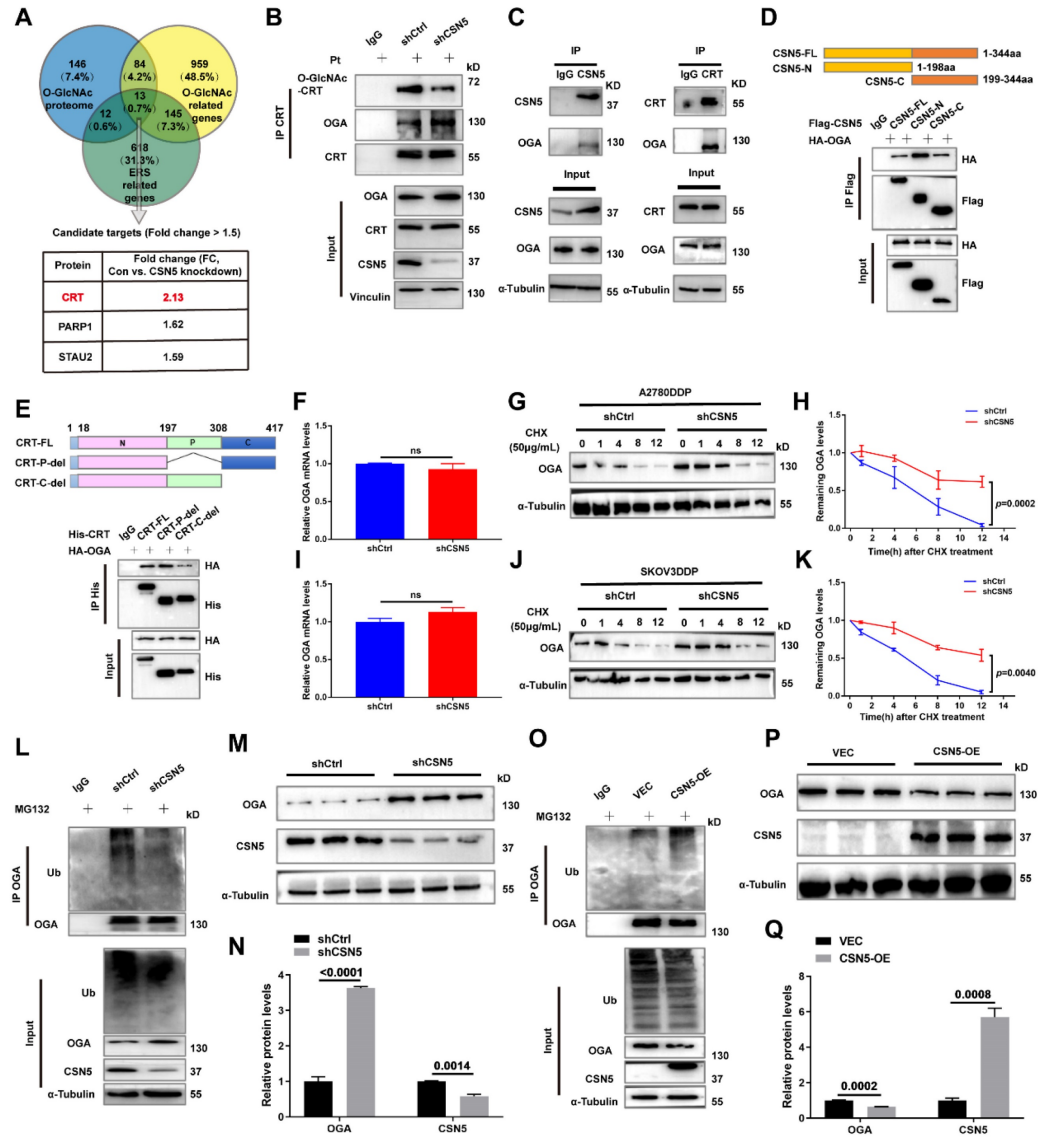
** CSN5 downregulation stabilizes OGA, modulating CRT O-GlcNAcylation.** (A) A Venn diagram was employed to show the co-expression of O-GlcNAc and ER stress-related genes (https://www.genecards.org/, accessed on May 2023). (B) A2780/DDP cells with or without CSN5 ablation were exposed to cisplatin (20 μM), followed by IP-IB analyses with the indicated antibodies. O-GlcNAcylated CRT and the combination of CRT to OGA were detected. (C) Total proteins of A2780/DDP cells were IP with anti-CSN5 (or anti-CRT) or normal IgG, followed by IB with the indicated antibodies. (D) HEK293T cells were transfected with Flag-CSN5 and HA-OGA in combination. IP-IB analyses were performed with the indicated antibodies after 24 h of transfection. (E) HEK293T cells were transfected with His-CRT and HA-OGA simultaneously. IP-IB analyses were performed with the indicated antibodies after 24 h of transfection. (F, I) DDP cells with or without CSN5 knockdown were exposed to cisplatin (20 μM) for 24 h, and the mRNA levels were determined by qRT-PCR. (G, H, J, K) DDP cells were treated with cycloheximide (CHX, 50 μM) for the indicated time, and the protein half-life time was evaluated by IB and quantified. (L) A2780/DDP cells with or without CSN5 inhibition exposed to cisplatin were treated with MG132 (20 μM) or vehicle control for 6 h before cell lysates, and ubiquitination levels of OGA were evidenced by IP-IB. (M) A2780/DDP cells were exposed to cisplatin (20 μM) for 24 h, and the related protein expression was evaluated by IB and quantified (N). (O) A2780/DDP-shCSN5 cells with or without CSN5 overexpression exposed to cisplatin were treated with MG132 (20 μM) or vehicle control for 6 h before cell lysates, and ubiquitination levels of OGA were evidenced by IP-IB. (P) A2780/DDP-shCSN5 cells with or without CSN5 overexpression were exposed to cisplatin (20 μM) for 24 h, and the related protein expression was evaluated by IB and quantified (Q).

In order to delineate the key O-GlcNAcylated modulator implicated in CSN5 inhibition-mediated apoptosis in platinum-resistant EOC cells, a Venn diagram was used to illustrate the co-expression of 13 genes among the O-GlcNAc proteome, O-GlcNAc, and ER stress-related genes. Calreticulin (CRT) was found to have changed the most significantly (**Figure 6A**). CRT O-GlcNAc levels were augmented in DDP cells with CSN5 overexpression relative to parental ovarian carcinoma cells but were diminished in CSN5 knockdown DDP cells, even though the overall CRT protein expression remained unchanged (**Figure 6B and Figure S6**). To further elucidate the interrelationship between CSN5, OGA, and CRT, Co-immunoprecipitation (Co-IP) was carried out. This revealed the endogenous co-interaction among OGA, CSN5, and CRT (**Figure 6C**). Additionally, HEK293T cells were transfected with Flag-CSN5 and HA-OGA, or His-CRT and HA-OGA, followed by reciprocal Co-IP assays. IB analysis using specific antibodies revealed that Flag-CSN5 co-immunoprecipitated with HA-OGA when both were co-expressed, with OGA primarily binding to the N-terminus of CSN5 (**Figure 6D**). Concurrently, deletion mutants of CRT were generated, and OGA was found to interact predominantly with the C-terminal of CRT (**Figure 6E**). Earlier findings showed that OGA protein expression increased in CSN5-deficient DDP cells, consistent with the well-documented role of CSN5 as a ubiquitination regulator 25, 26. Thus, it was speculated that CSN5 might influence the stability modulation of OGA. A Cycloheximide (CHX) chasing assay unveiled an enhancement in OGA protein stability following CSN5 downregulation, without impacting OGA mRNA levels, indicating that CSN5 affects OGA levels post-translationally (**Figure 6F-K**). Furthermore, it was corroborated that ubiquitination of OGA was reduced and protein expression was augmented following CSN5 ablation (**Figure 6L-N**). Conversely, exogenous overexpression of CSN5 in A2780/DDP cells with stable CSN5 knockdown increased OGA ubiquitination and depressed OGA expression in comparison to vector control (**Figure 6O-Q**). Collectively, these data suggest that CSN5 may modulate CRT O-GlcNAcylation by regulating OGA protein stability.

### 3.7 O-GlcNAcylation of CRT at T346 confers EOC cells with tolerance to platinum-induced cell death

**Figure 7:**
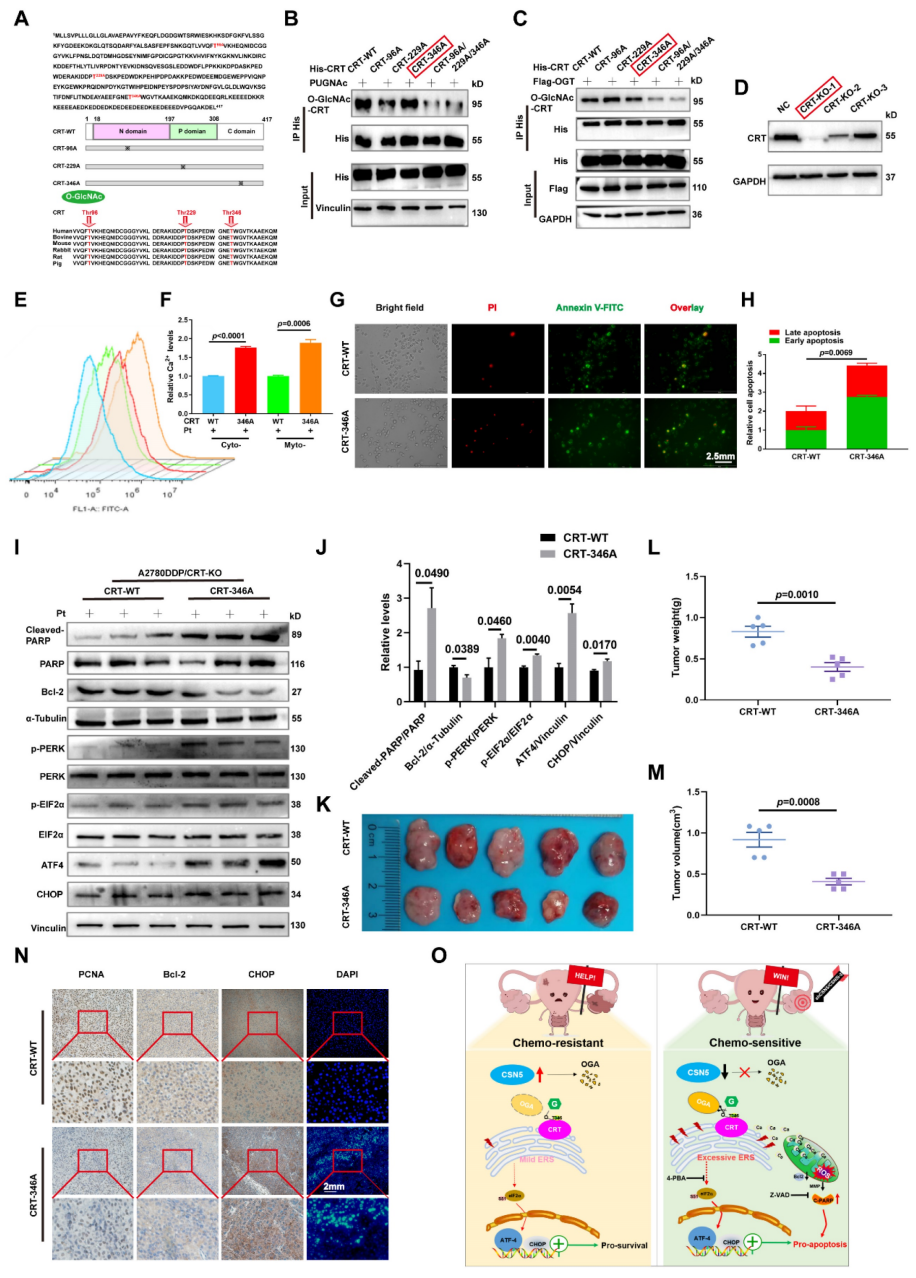
** O-GlcNAcylation of CRT at T346 confers EOC cells with tolerance to platinum-induced cell death.** (A) Potential O-GlcNAc modified sites of CRT were identified by IP-MS, and conservation analysis was performed. (B) Flag-tagged point mutation plasmids were constructed for all potential O-GlcNAcylated sites of CRT, and HEK293T cells were transfected with indicated point mutants of CRT for 24 h. Then, HEK293T-CRT-WT and alanine-substituted CRT cells were treated with PUGNAc (an inhibitor of OGA, 25 μM) for another 24 h followed by IP-IB analysis. (C) HEK293T cells were transfected with indicated point mutants of CRT accompanied by Flag-OGT for 24 h. Then, the protein expression levels and the O-GlcNAcylation status were analyzed by IP-IB. (D) Validation of the CRT knock out efficiency in HEK293T cells by IB. (E) CRT-KO A2780/DDP cells stably expressing His-CRT (WT or T346A) were treated with cisplatin (20 μM) for 24 h, and then subjected to FCM analysis of Ca^2+^. (F) The levels of Ca^2+^ were statistically analyzed and shown as mean ± SEM. (G) Cellular apoptosis was examined by immunofluorescent staining in CRT-KO A2780/DDP cells stably expressing His-CRT (WT or T346A) after the treatment with cisplatin (20 μM) for 24 h (Scale bar = 2.5 mm), and the ratio of apoptotic cells was quantified and statistically analyzed (H). (I) The apoptotisis and ER stress markers were detected by IB, and the relative protein quantification was performed and statistically analyzed (J). (K) In vivo xenograft model showed the tumor suppressive effects of CRT T346A mutant. (L, M) Tumor volume and weight were analyzed. (N) IHC results exhibited the expression of the proliferative marker PCNA, the anti-apoptotic marker Bcl-2, and ER stress marker CHOP. Apoptotic cells were displayed via DAPI staining (Scale bar = 2 mm). (O) Schematic illustration depicting the significance of the CSN5/CRT O-GlcNAc/ER stress axis in platinum-resistant ovarian carcinoma.

The prior data revealed that CRT's O-GlcNAc was influenced by OGA, which was regulated by CSN5 in DDP cells. To identify the potential O-GlcNAcylation sites on CRT, MS was utilized for more refined examination. Multiple possible O-GlcNAc modification locations, including T96, T229, and T346, were identified (**Figure S7**). Sequence alignment showed that the residues T96, T229, and T346 were located within CRT's N domain (residues 18-197), P domain (residues 198-308), and C domain (residues 309-417), respectively, and these were found to be highly conserved across various species (**Figure 7A**).

To verify the principal O-GlcNAcylated site on CRT, point mutation plasmids were created using single amino acid substitution from threonine (T) to alanine (A) with His-tags. Wild type or point mutants of CRT was introduced into HEK293T cells for 24 h, followed by treatment with the OGA inhibitor PUGNAc for another 24 h. The modification of CRT by O-GlcNAc was observed using IB with an anti-O-GlcNAc antibody after His IP. Notably, all mutants except CRT T346A were found to be unmodifiable by O-GlcNAc after OGA inhibition, suggesting that the T346 site might be the primary O-GlcNAc modification site (**Figure 7B).** This was further corroborated by experiments involving OGT overexpression with point mutants (**Figure 7C**). To further explore the role of O-GlcNAc-CRT at T346 in CSN5-mediated platinum resistance of ovarian carcinoma, CRT deletion cell lines were generated and confirmed using CRISPR/Cas9 and IB (**Figure 7D**). Subsequently, His-CRT (WT or T346A) expression plasmids were transfected into CRT-KO A2780/DDP cells and exposed to cisplatin. Functional assays depicted that both the aggregation of Ca^2+^ in the cytoplasm and mitochondria, and the apoptosis potential in CRT-T346A mutants were more prominent than in the CRT-WT counterparts in CRT-KO A2780/DDP cells (**Figure 7E-H**). Additionally, the apoptotic marker cleaved-PARP and the ER stress markers including p-PERK/PERK, p-EIF2α/EIF2α, ATF4 and CHOP increased, while the anti-apoptotic protein Bcl-2 decreased in CRT-KO A2780/DDP cells following CRT-T346A mutant transfection (**Figure 7I, J**). In vivo xenograft studies further provided evidence that CSN5-overexpressing 2780/DDP cells with CRT-T346A mutant transfection exhibited smaller tumor size and weight compared to their counterparts (**Figure 7K-M**). IHC staining revealed that both the proliferative marker PCNA and the anti-apoptotic maker Bcl-2 were reduced, while ER stress signaling molecule CHOP and apoptosis, as indicated by DAPI staining, increased in tissues of mice bearing tumors with CRT-T346A mutants (**Figure 7N**). In summary, these findings demonstrate that the inhibition of T346 O-GlcNAc-CRT either by CSN5 deficiency or CRT T346A mutation augments the sensitivity of DDP cells to platinum therapy. Specifically, in ovarian cancer cells resistant to platinum treatment, overexpressed CSN5 maintains CRT O-GlcNAc at a heightened level by destabilizing OGA, which stimulates a cytoprotective UPR to prevent the platinum-resistant EOC cells from chemotherapeutic killing (**Figure 7O**).

## 4. Discussion

In this study, an integrated analysis of RNA-sequencing and O-GlcNAc proteomics has revealed that CRT O-GlcNAcylation and ER stress (encompassed by ATF4-CHOP signaling) are instrumental in CSN5-mediated platinum resistance in EOC. Specifically, O-GlcNAcylation of CRT at the T346 position is removed by OGA in reaction to CSN5 suppression. This reduction in O-GlcNAcylated CRT leads to a deficiency in Ca^2+^ buffering, thereby serving as the initiating factor for ER stress signaling. Additionally, the subsequent aggregation of Ca^2+^ in the cytoplasm and mitochondria amplifies this signaling pathway, culminating in the induction of apoptosis in platinum-resistant EOC cells. Conversely, in EOC cells that exhibit resistance to platinum therapy, overexpression of CSN5 maintains CRT O-GlcNAc at an elevated level by destabilizing OGA. This leads to the activation of moderate UPR responses, thereby conferring the EOC cells with increased resilience to platinum-induced cell death (**Figure 7O**).

In our laboratory, CSN5 has been identified as upregulated in ERα-positive breast cancer, leading to tamoxifen resistance 26. Subsequent studies have revealed that CSN5 initiates the occurrence and development of ovarian carcinoma 27, and it is further upregulated in platinum-resistant EOC tissues and in vitro DDP cells 16. In addition, lentivirus-mediated CSN5 downregulation can effectively re-sensitize DDP cells to platinum treatment 16. However, the specific mechanisms underlying CSN5 inhibition-mediated platinum sensitivity in EOC remain unclear. In the present study, both in vitro and in vivo tumor growth were inhibited, and cellular apoptosis was elevated following CSN5 inhibition. With advances in genome sequencing, focus on the modification and functional identification of proteins has increased 28, 29. The differentially expressed genes, as revealed by transcriptome sequencing in DDP cells with CSN5 knockdown, were concentrated in protein translational modification (PTM) and proteoglycans in cancer. PTMs are well-established as a central mechanism extending beyond the genetic code, modulating cellular function in response to developmental or environmental stimuli 30. Specific PTMs such as glycosylation, phosphorylation, acetylation, and ubiquitination are deeply involved in tumorigenesis and immune modulation and are emerging as crucial targets for early detection and cancer treatment 31-33. Therefore, levels of glycosylation (O-GlcNAcylation), phosphorylation, acetylation, and ubiquitination across broad-spectrum proteins were examined. Protein O-GlcNAcylation was found to be significantly reduced in CSN5-deficient DDP cells, suggesting that abnormal O-GlcNAcylation of essential regulatory proteins may be potential reasons for EOC platinum resistance.

Since protein O-GlcNAcylation is primarily regulated by OGT, O-GlcNAcase (OGA), and glutamine-fructose-6-phosphate aminotransferase 1 (GFPT1), the rate-limiting enzyme in the HBP. We further explored the relationships between CSN5 and O-GlcNAc-related modulators. Our results indicated an upregulated expression of OGA, while levels of OGT and GFPT1 remained unchanged. In a clinical context, OGA was negatively correlated, and protein O-GlcNAc was positively correlated with CSN5 expression in EOC patients. Moreover, employing O-GlcNAc proteomics uncovered significant enrichment of differential O-GlcNAc proteins in the ER, associated with ER stress and apoptosis-related signaling pathways. ER stress, typically accompanied by UPR, mainly activates three classical pathways including inositol-requiring enzyme 1 (IRE1-XBP1), protein kinase RNA-like endoplasmic reticulum kinase (PERK-ATF4-CHOP), and activation transcription factor 6 (ATF6) pathway 34. Further qRT-PCR and IB results showed no significant changes in the expression of IRE1 and ATF6 signaling proteins, but the ATF4-CHOP pathway, downstream of PERK, was notably activated after CSN5 suppression. This finding underscores the involvement of ER stress-related ATF4-CHOP signaling in the platinum resistance of EOC.

Accumulating evidence highlights that Ca^2+^ signaling serves a critical role in stress responses, as Ca^2+^ participates in numerous cellular activities 35, 36. Predominantly localized within the ER, disturbances in Ca^2+^ have been demonstrated to trigger ER stress 37, 38. In this context, cellular Ca^2+^ levels were analyzed to assess whether CSN5 ablation might induce ER stress through the disruption of Ca^2+^ homeostasis. As anticipated, concentrations of Ca^2+^ in the cytoplasm and mitochondria increased following CSN5 inhibition either genetically or pharmacologically. This effect was furtherly augmented by the presence of cisplatin. Overloading of Ca^2+^ can lead to mitochondrial dysfunction, thereby enhancing ROS generation through metabolic pathways and subsequently impairing Ca^2+^ transport systems 39, 40. In alignment with prior findings, the translocation of Ca^2+^ into the mitochondria caused by CSN5 inhibition further precipitated mitochondrial dysfunction, as evidenced by reduced MMP and increased ROS production. Roberta et al. revealed that Ca^2+^ signaling is an initiating step in cisplatin-induced apoptosis 41. However, cisplatin-mediated apoptosis can be negated after the development of platinum resistance in ovarian carcinoma. In the present study, both genetic and pharmacological suppression of CSN5 enhanced the cellular apoptosis of DDP cells, an effect that was amplified with cisplatin treatment. In addition to apoptosis, ferroptosis has been recognized as being triggered by oxidative stress 42. Nonetheless, an examination of ferroptosis-related markers revealed no significant difference between the CSN5 downregulation and the control group (**Figure S8**).

In our observations, DDP cells exhibited basal activation of ER stress relative to their corresponding parental counterparts, potentially conferring DDP cells with tolerance to cisplatin-induced cell death (**Figure S9**). Conversely, inhibition of CSN5 in platinum-resistant EOC cells further escalated the ER stress signaling pathway, as evidenced by a significantly upregulated ATF4-CHOP pathway protein expression and an increase in apoptosis. ATF4-CHOP signaling was identified as a cytoprotective pathway, independent of other parallel stress-elicited signals; its activation could promote a stress-resistant preconditioned status 43. Our study revealed that re-overexpression of CSN5 in CSN5-deleted DDP cells negated the upregulation of ATF4-CHOP pathway protein expression, mitigated apoptosis, and restored cell viability (**Figure S10**). Additionally, alterations in the protein folding environment (such as hypoxia, drug exposure, high glucose levels, nutrient deficiency, low pH, genetic mutation, and Ca^2+^ homeostasis imbalance) may lead to the aggregation of unfolded or misfolded proteins, thereby causing ER stress and initiating UPR to alleviate protein misfolding and re-establish homeostasis, preserving cell survival 9 (Mild UPR). If the stress intensity continues unabated or becomes over-activated, it may substantially trigger the apoptotic signaling pathway in cells, culminating in cellular fatality 10, 11 (Terminal UPR). These findings suggest that CSN5 might orchestrate the balance of ER stress in the presence of platinum-based drugs, where mild ER UPR signals could be sustained to counter the cytotoxic effects of platinum-based drugs on EOCs, thus contributing to the development of drug resistance.

Reportedly, CHOP, functioning as a transcription factor, can directly instigate ER stress-mediated apoptosis 44. The introduction of 4-PBA (an ER stress signaling inhibitor) or the application of small siRNA-mediated CHOP knockdown alleviated, though did not fully reverse, the cellular apoptosis brought on by CSN5 inhibition, especially when combined with cisplatin. This effect was further corroborated in an in vivo xenografted mouse model where mice bearing tumors with CSN5 deletion and 4-PBA treatment exhibited larger tumor sizes compared to those with only CSN5 knockdown, yet smaller than the control group. These findings suggest that CSN5 suppression may partially trigger cellular apoptosis via the direct activation of the ATF4-CHOP pathway. In addition, beyond the apoptosis induced by ER stress, excessive mitochondrial ROS production may precipitate caspase-dependent cellular destruction 45. This implies that CSN5 intervention might concurrently induce cell apoptosis through two pathways. As expected, cellular apoptosis resulting from CSN5 inhibition could be negated following Z-VAD-FMK (a pan-caspase inhibitor) pretreatment (**Figure S5E, F**). Collectively, these outcomes demonstrate that CSN5 ablation combined with cisplatin amplifies cellular chemosensitivity through disruptions in Ca^2+^ homeostasis, culminating in both caspase-dependent and independent cellular apoptosis. This reveals a pivotal role for Ca^2+^ homeostasis in the CSN5-mediated platinum sensitivity of OC. Future research must explore how CSN5 reduction leads to imbalances in Ca^2+^ homeostasis.

The role of aberrant protein O-GlcNAc-related ER stress in CSN5-mediated platinum resistance of DDP cells underscores the need to pinpoint the crucial O-GlcNAc player in this process. Utilizing O-GlcNAc proteomics, CRT emerged as the potential contributor to the ER stress instigated by CSN5 inhibition in DDP cells. Within the ER, Ca^2+^ is mainly buffered by CRT 46, recognized as the principal soluble Ca^2+^-binding glycoprotein in the ER. Furthermore, CRT can be activated in response to ER stress 14. This suggests that CRT may be involved in CSN5-mediated homeostasis disequilibrium of ER stress, culminating in cellular apoptosis. An examination of CRT O-GlcNAc levels revealed an upregulation in the DDP cells compared to the corresponding parental ones (**Figure S6**). Concurrently, CRT O-GlcNAc was found to be attenuated in CSN5 knockdown DDP cells, without altering CRT's protein expression levels (**Figure 6B and Figure S6D**). A series of endogenous and exogenous experiments revealed an interaction between CSN5 and OGA, with OGA primarily interplaying with CRT's C-terminal, a domain responsible for Ca^2+^ binding. Intriguingly, given CSN5's previously reported role in protein ubiquitination 25, 47, 48, it was postulated that CSN5 might influence the regulation of OGA protein stability, consequently affecting CRT's O-GlcNAc. This hypothesis was supported by results showing increased protein stability of OGA and reduced ubiquitination following CSN5 downregulation in DDP cells. In contrast, conflicting results were noted upon re-introduction of CSN5 in CSN5 deletion DDP cells, affirming CSN5's mediation of OGA protein stability. Further analysis to identify potential O-GlcNAc sites in CRT utilized IP-MS, suggesting T96, T229, and T346 of CRT as candidate modification sites (**Figure S7**). Subsequent experimentation confirmed that mutation of CRT-O-GlcNAc at T346 disrupted Ca^2+^ homeostasis and increased apoptotic sensitivity in DDP cells. This unveiled that O-GlcNAcylation of CRT at T346 is vital for the survival of platinum-resistant EOC cells in a platinum environment. Collectively, these findings illuminate the significant role of the CSN5/CRT O-GlcNAc/ER stress regulatory axis in determining the chemosensitivity of EOC.

In summary, the inhibition of CSN5-mediated CRT O-GlcNAc decay at T346 mitigates the resistance of EOCs to ER stress injury under platinum conditions. These findings unveil a novel regulatory axis involving CSN5/CRT O-GlcNAc/ER stress in platinum-resistant ovarian carcinoma. Furthermore, it provides new insights into an effective approach to resolving platinum chemo-resistance by targeting the CSN5/CRT O-GlcNAc/ER stress signaling pathway.

## Supplementary Material

Supplementary figures and tables.

## Abbreviations

CSN5: COP9 signalosome subunit 5
EOC: epithelial ovarian cancer
ER: endoplasmic reticulum
CRT: calreticulin
DDP: cisplatin
OGT: O-GlcNAc transferase
OGA: O-GlcNAcase
ROS: reactive oxygen species
MMP: mitochondrial membrane potential
DEGs: differential expression genes
PTM: post translational modification
GO: Gene oncology
KEGG: Kyoto Encyclopaedia of Genes and Genomes
IP-IB: immunoprecipitation-immunoblotting
IF: immunofluorescence
IHC: immunohistochemistry
MS: mass spectrometry
FCM: flow cytometry
CHX: Cycloheximide
