# Supplementary material for: A novel CSN5/CRT O-GlcNAc/ER stress regulatory axis in platinum resistance of epithelial ovarian cancer
Source: Int J Biol Sci. 2024 Jan 27;20(4):1279–96. doi: 10.7150/ijbs.89700 (PMC10878149; doi:10.7150/ijbs.89700)
Supplement: Supplementary file 1 — Supplementary figures and tables. [file ijbsv20p1279s1.pdf]

Figure S1

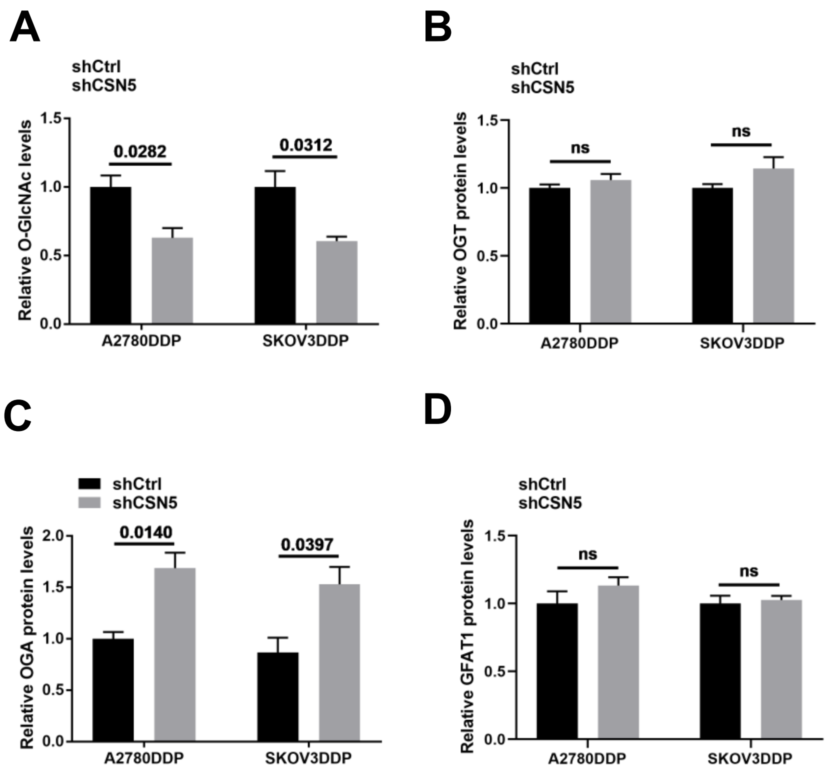

Figure S1. Statistic analyses of protein expression in DDP cells with or without CSN5 knockdown. Data are presented as mean ± SEM.

Figure S2

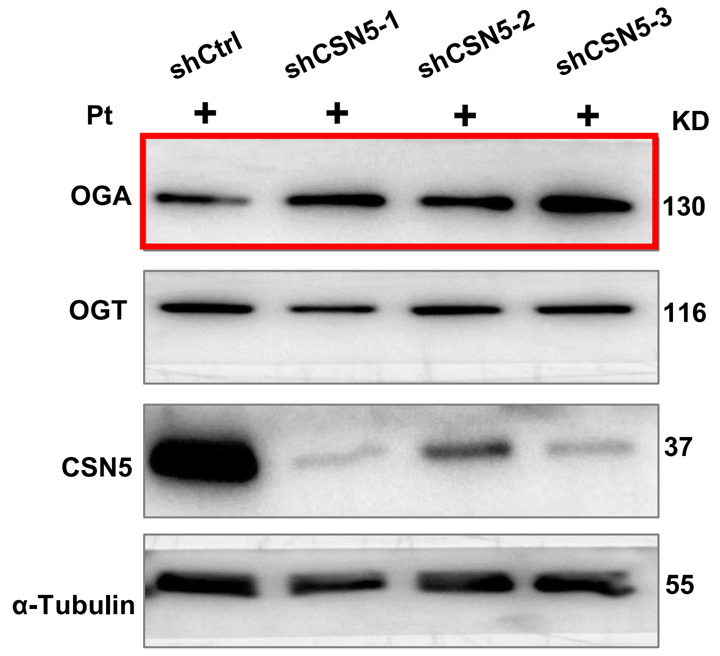

Figure S2. Effects of CSN5 knockdown on the expression of OGA and OGT.

Figure S3

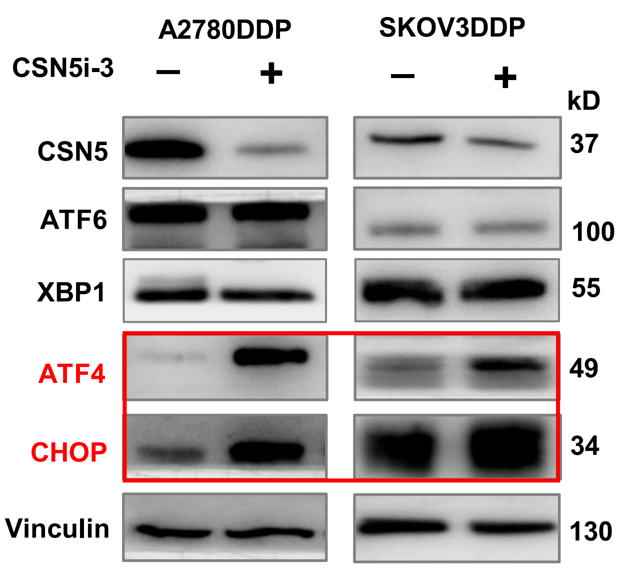

Figure S3. Effects of CSN5 knockdown on the expression of ER stress markers.

Figure S4

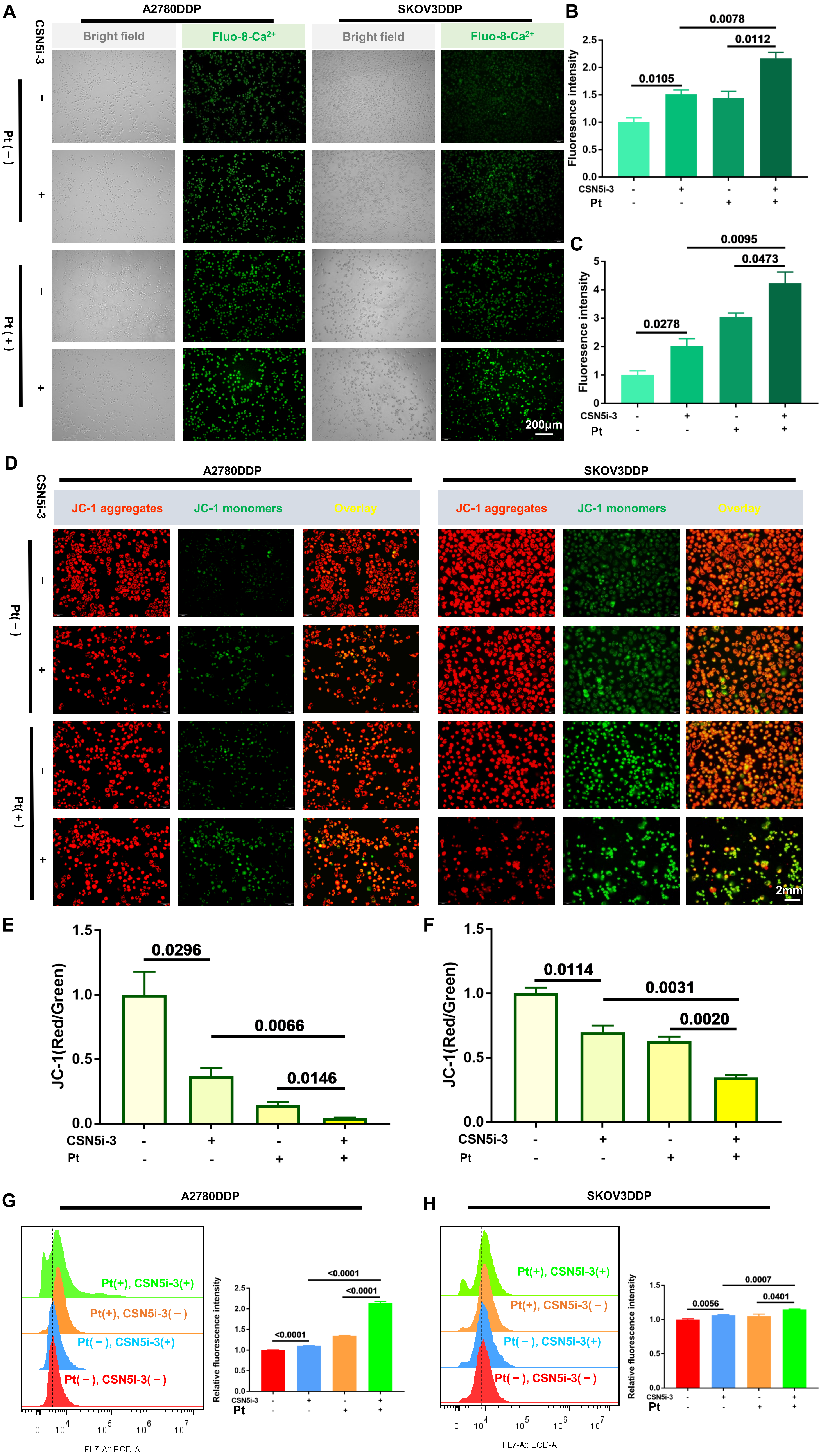

Figure S4. CSN5 inhibition induced Ca^{2+} disturbances and mitochondrial dysfunction. A2780/DDP and SKOV3/DDP cells with or without CSN5 knockdown were treated with cisplatin for 24 h. (A-C) Immunofluorescent staining was used to examine cytoplasmic Ca^{2+} levels (Scale bar = 200 μm). (D) The disruption of MMP was investigated using JC-1 assay by immunofluorescent staining (Scale bar = 2 mm). (E, F) Relative JC-1 red/green ratio is represented as a bar graph in percentage-ratio under the Immunofluorescent staining data. (G, H) ROS levels were investigated using DHE labeled kit. Relative cellular ROS levels were represented as a bar graph in percentage-ratio under the flow cytometry data. All experiments were performed in triplicate.

Figure S5

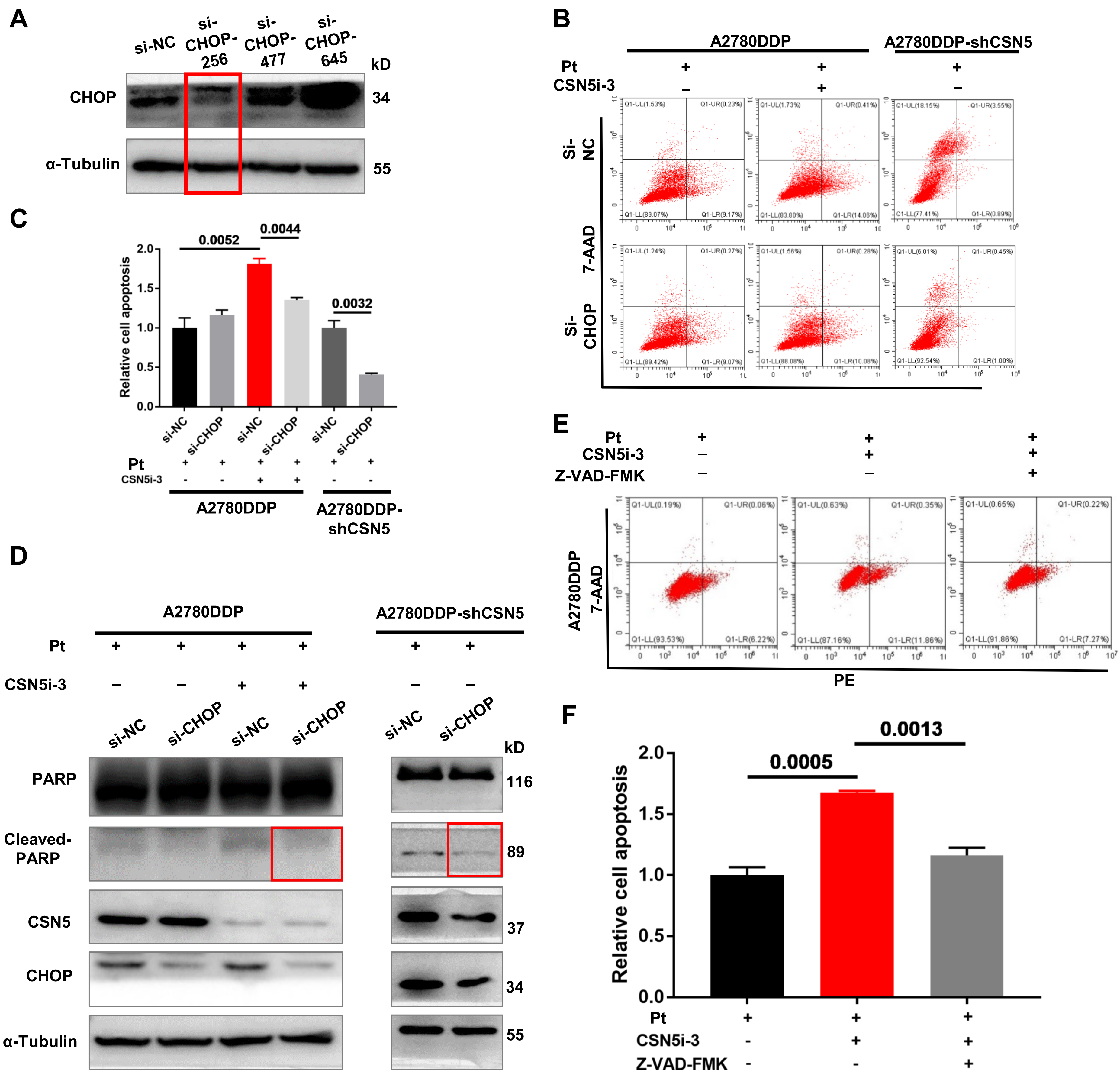

Figure S5. Genetic inhibition of CHOP or caspase suppressant involvement partly abrogated the apoptosis induced by CSN5 inhibition. (A) Knockdown efficiency of CHOP in A2780/DDP cells was evaluated by Western blot. (B) A2780/DDP cells with or without CSN5 inhibition were exposed to cisplatin for 24 h, and FCM was utilized to detect the apoptotic ratio. (C) The apoptotic ratio were quantified by histogram. (D) A2780/DDP cells with or without CSN5 inhibition were exposed to cisplatin for 48 h, and the apoptotic markers Cleaved-PARP/PARP were analyzed by Western blot. (E) A2780/DDP cells with or without CSN5 inhibition pretreated with pretreatment of pan-caspase suppressor(Z-VAD-FMK, 10 μM) or not were exposed to cisplatin for 24 h, and flow cytometry analysis was utilized to detect the apoptotic ratio. (F) The apoptotic ratio was quantified as mean ± SEM.

Figure S6

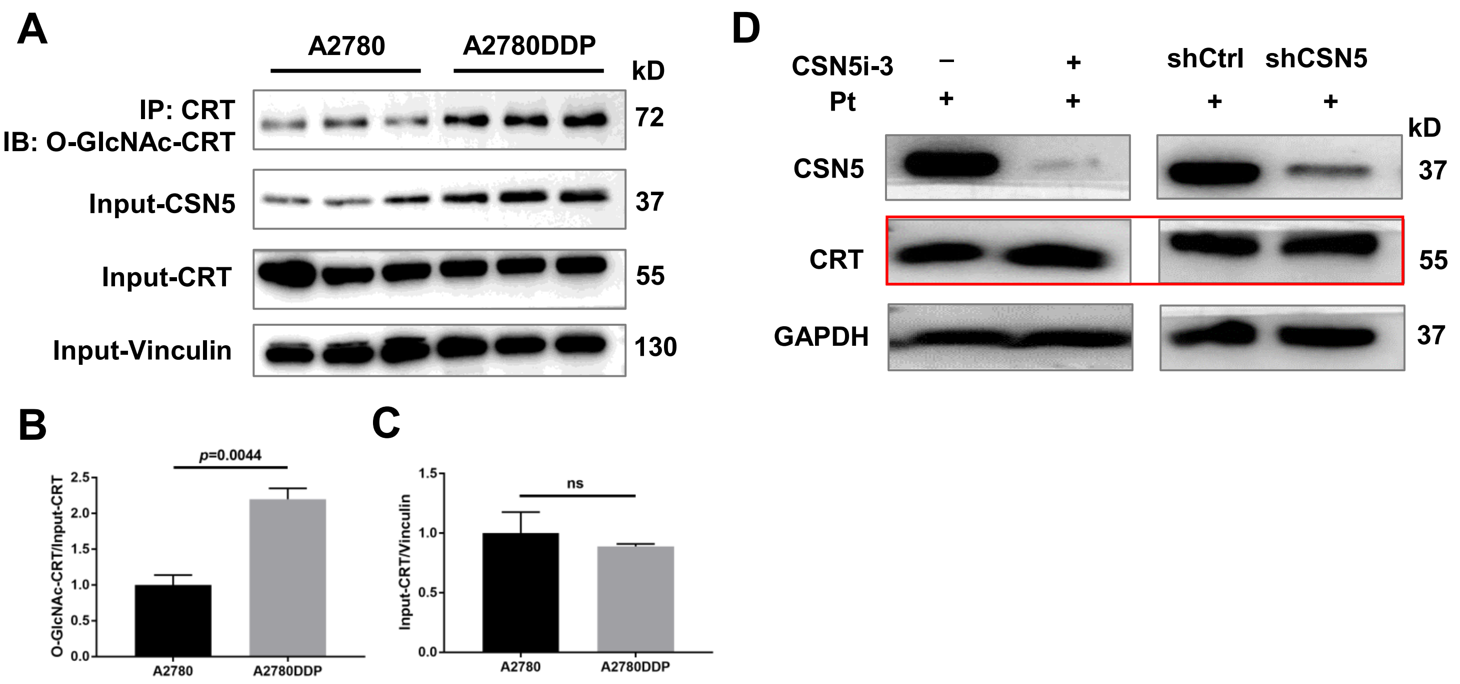

Figure S6 Levels of CRT and its O-GlcNAc in ovarian cancer parent cell 2780 and platinum-resistant cell A2780/DDP with or without CSN5 downregulation. (A) Expression levels of O-GlcNAcylated CRT in ovarian cancer parent cell 2780 and platinum-resistant cell A2780/DDP was detected by Immunoprecipitation and Immunoblotting (IP-IB) with the indicated antibodies. (B) The ratio of O-GlcNAc-CRT to total CRT was quantified. (C) The ratio of total CRT to internal reference protein vinculin was quantified. (D) Expression levels of CRT in A2780/DDP cells with or without CSN5 inhibition was detected by IB.

Figure S7

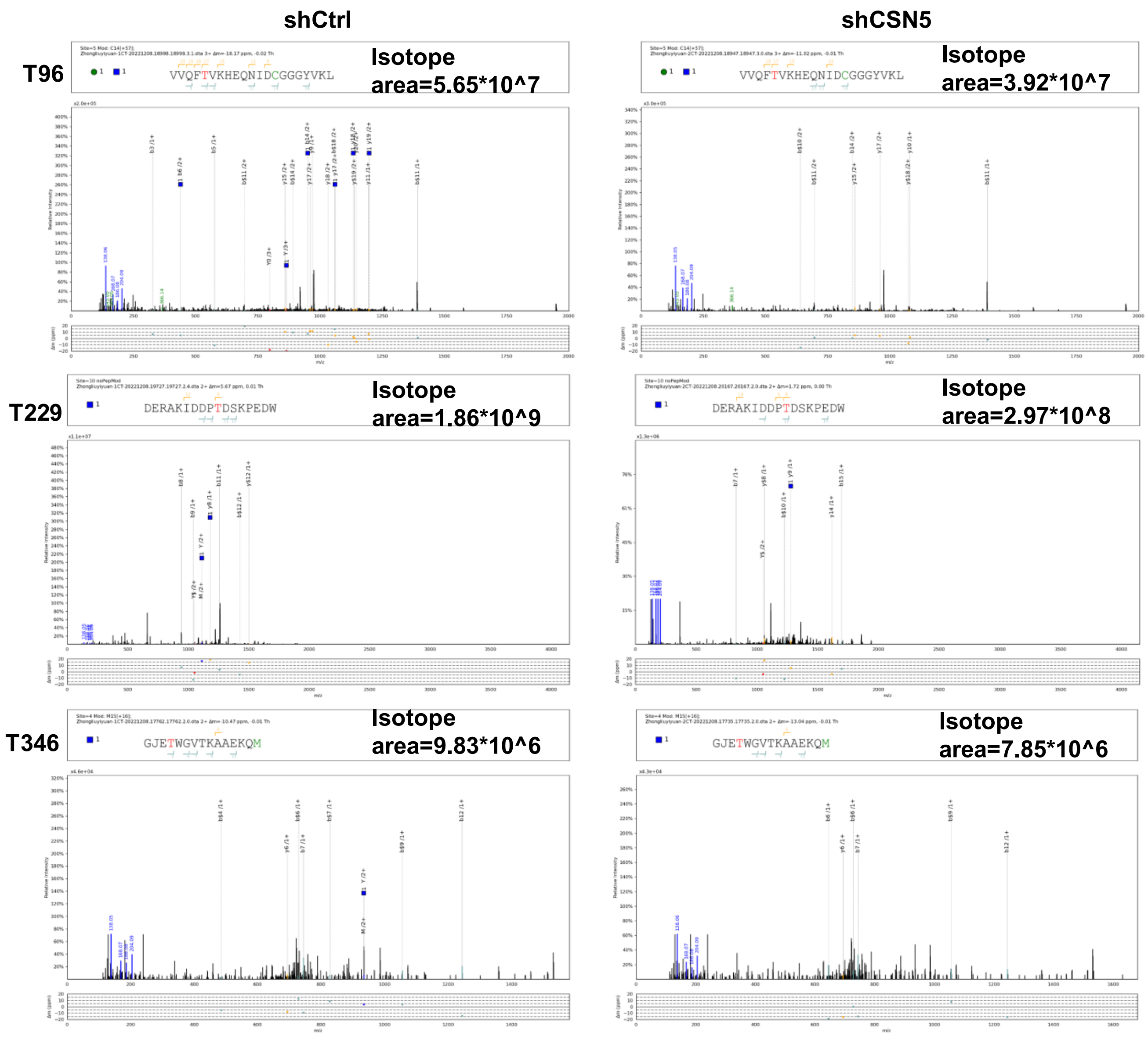

Figure S7. The potential O-GlcNAcylation sites screened by the LC-MS/MS.
CRT was purified from A2780/DDP cells with or without CSN5 knockdown, and analyzed by MS to identify the O-GlcNAcylation sites. Three different O-GlcNAcylation sites in CRT were shown.

Figure S8

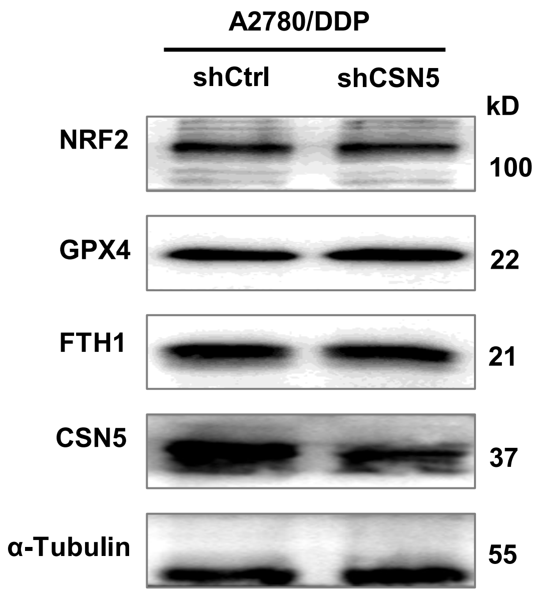

Figure S8 Levels of ferroptosis-related markers in A2780/DDP cells with or without CSN5 knockdown.

Figure S9

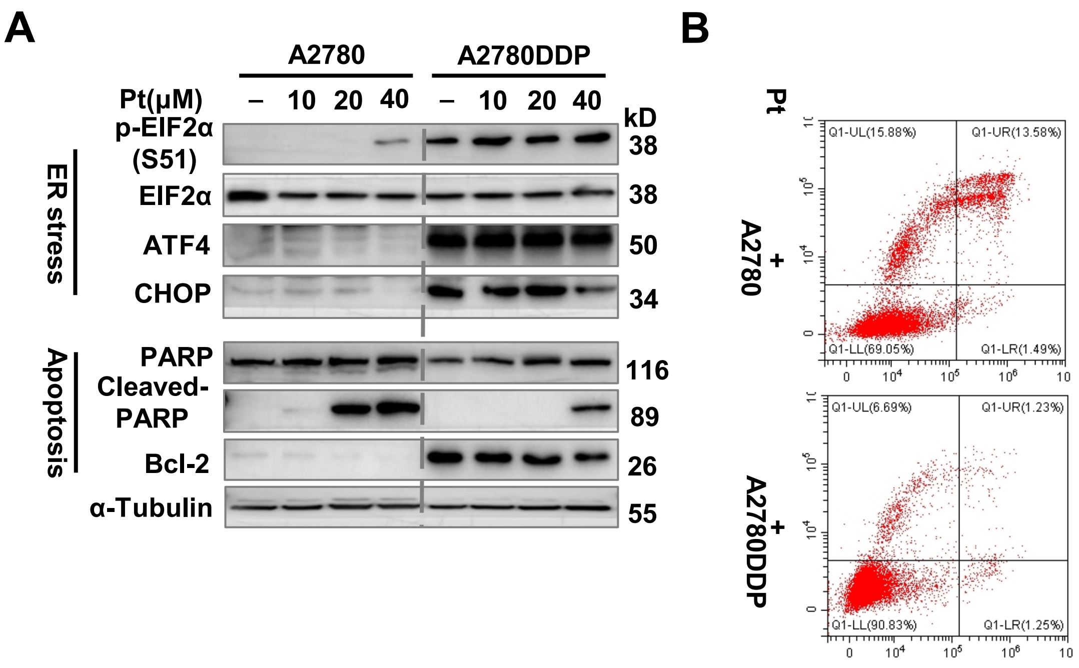

Figure S9. Basal activation of ER stress endowed DDP cells with tolerance to cisplatin-induced cell death. (A) Parental OC cells and the corresponding platinum-resistant ones were exposed to cisplatin in different doses, and the ER stress and apoptotic markers were detected by IB. (B) Parental EOC cells and the corresponding DDP-resistant ones were treated with cisplatin for 24 h, and the apoptotic ratio were tested by flow cytometry analysis.

Figure S10

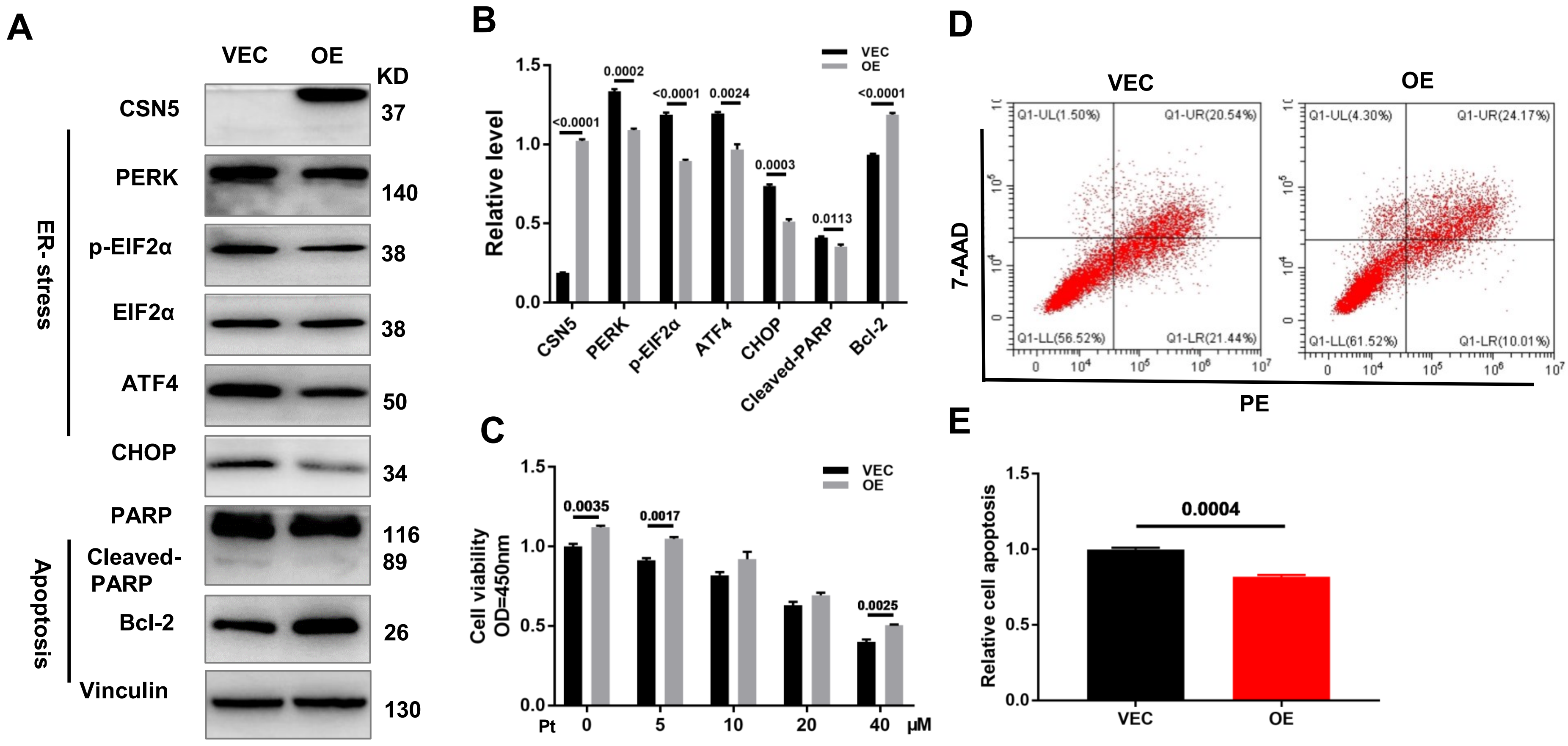

Figure S10. Re-overexpression of CSN5 in CSN5 knockdown A2780/DDP cells inhibited the ER stress signaling and stimulated platinum-resistant phenotypes. (A, B) ER stress and apoptosis-related markers were illustrated by IB, and the protein gray value was statistically quantified as mean ± SEM. (C) Cell viability was detected by CCK-8 assay. (D, E) Cell apoptosis was evaluated via flow cytometry analysis and the relative apoptotic ratio was quantified as mean ± SEM.

Table S1 shRNA sequences.

Targets	Sequence 5’-3’
shCSN5-1	CGCCTTTAGGACATACCCAAA
shCSN5-2	GTCTCAGGTTATTAAGGATAA
shCSN5-3	GATCCGCTTACGAATGCAGACTATAC
shCtrl	CCTAAGGTTAAGTCGCCCTCG

Table S2 siRNA sequences.

Targets		Sequence 5’-3’
siCHOP-256	F	GGUCCUGUCUUCAGAUGAATT
	R	UUCAUCUGAAGACAGGACCTT
siCHOP-477	F	GAACCAGGAAACGGAAACATT
	R	UGUUUCCGUUUCCUGGUUCTT
siCHOP-645	F	GAGCUCUGAUUGACCGAAUTT
	R	AUUCGGUCAAUCAGAGCUCTT
siNC	F	UGACCUCAACUACAUGGUUTT
	R	AACCAUGUAGUUGAGGUCATT

Table S3 Antibodies used in this study.

Antibody	Manufacturer	Code number
CSN5	CST	6895
CRT	Santa Cruz	SC-373863
CRT	Abcam	92516
PERK	CST	5683
p-EIF2 α	CST	3398
EIF2 α	CST	5324
ATF4	Proteintech	10835-1-AP
CHOP	Proteintech	15204-1-AP
ATF6	Proteintech	24169-1-AP
XBP1	Santa Cruz	SC-8015
ATF3	CST	18665
PARP	CST	9532
Cleaved-PARP	CST	5625
Bcl-2	Proteintech	12789-1-AP
Cyt C	CST	4280
PCNA	Santa Cruz	SC-56
O-GlcNAc	Abcam	2739
O-GlcNAc	Abcam	202665
OGA	Abcam	124807
OGT	Abcam	177941
GFPT-1	Proteintech	14132-1-AP
Vinculin	CST	13901
Tubulin	Proteintech	11224-1-AP
GAPDH	Proteintech	60004-1-Ig
Phos (Ser/Thr)	AmyJet Scientific	PP2551
Phos (Tyr)	PTM BioLab	PTM-702RM
Acetylation	PTM BioLab	PTM-105RM
Ubiquitination	Santa Cruz	SC-166553
Flag-tag	CST	14793
Flag-tag	Proteintech	66008-4-Ig
His-tag	CST	12698
His-tag	Proteintech	10001-0-AP
His-tag	Proteintech	66005-1-Ig
HA-tag	Proteintech	51064-2-AP
NRF2	CST	12721
GPX4	Proteintech	67763-1-Ig
FTH1	CST	4393
HRP-conjugated Goat Anti-Rabbit IgG	Proteintech	SA00001-2
HRP-conjugated Goat Anti-Mouse IgG	Proteintech	SA00001-1
Goat anti-Rabbit IgG, DyLight™ 488	Invitrogen	35552
Goat anti-Mouse IgG, Alexa Fluor™ 555	Invitrogen	A32727
Universal anti-Mouse/Rabbit-HRP	Long Island Antibody	D-3004

Table S4 CRT-CRISPR/Cas9-sgRNA target sequences.

NO.	TargetSeq
sgRNA-1	GCCGCACAATCAGTGTGTAC
sgRNA-2	GAAGATGACATGAACCTTCT
sgRNA-3	CTCAGTTCCGGCAAGTTCTA

Table S5 Primers for qRT-PCR.

Genes		Primer sequence 5’-3’
Csn5	F	GATCGGGAGGCAACTTGGAAG
	R	TGCGGATATTGTTCTTGTTGGA
Atf4	F	CATTCCTCGATTC CAGCAAAGCAC
	R	TTCTCCAACATCCAATCTGTCCCG
chop	F	GCACCTCCCAGAGCCCTCACTCTCC
	R	GTCTACTCCAAGCCTTCCCCCTGCG
Atf3	F	TGGCAACACGGAGTAAACGA
	R	GCATCATTTTGCTCCAGGCTC
Atf6	F	AACAAGACCACAAGACCA
	R	AGGAGGAACTGACGAACT
Xbp1	F	AGAGTAGCAGCTCAGACTGC
	R	CATTAATGGCTTCCAGCTTG
gapdh	F	TGTTCGTCATGGGTGTGAACC
	R	GCAGTGATGGCATGGACTGTG
